# Supplementary material for: Polygenic prediction of occupational status GWAS elucidates genetic and environmental interplay in intergenerational transmission, careers and health in UK Biobank
Source: Nat Hum Behav. 2024 Dec 23;9(2):391–405. doi: 10.1038/s41562-024-02076-3 (PMC11860221; doi:10.1038/s41562-024-02076-3)
Supplement: Supplementary file 1 — FAQ, Research Plan, Supplementary Background Information, Analyses, Discussion, Figs. 1–16, and Tables 1–16. [file 41562_2024_2076_MOESM1_ESM.pdf]

nature human behaviour

Supplementary information https://doi.org/10.1038/s41562-024-02076-3

Polygenic prediction of occupational status GWAS elucidates genetic and environmental interplay in intergenerational transmission, careers and health in UK Biobank

In the format provided by the authors and unedited

Table of Contents

List of supplementary figures 3

List of supplementary tables 4

1. Frequently Asked Questions (FAQ) 5

2. Background 17

3. Measuring occupational status and prestige 17

4. Research plan 18

5. Phenotype definitions 18

6. Representativity of the UK Biobank with the Office of National Statistics (ONS) 18

7. Overview of GWAS analyses 20
   - 7.1 Analyses 20
   - 7.2 Sample inclusion criteria 20
   - 7.3 Findings 21
   - 7.4 Replication 21

8. SNP-heritability 22

9. Population stratification test 23

10. Polygenic score calculation and prediction 24
   - 10.1 Calculation of polygenic scores 24
   - 10.2 Out-of-sample prediction 24

11. Uncovering genetic communality of occupational status and prestige with socio-economic and other measures using Genomic SEM 24
   - 11.1 General factor of occupational status 24
   - 11.2 General factor of socioeconomic status (SES) 26
   - 11.3 Mediators between polygenic signals and occupational status 28

12. Direct and indirect effects 31
   - 12.1 Parental Control design 31
   - 12.2 Adoption design 31
   - 12.3 Sibling design 31
   - 12.4 The sibling design and assortative mating 32

13. Polygenic score prediction over the life course 34

14. Polygenic scores and the intergenerational transmission of occupational status 36

15. Polygenic score mediation by occupational aspirations and psychological traits 37
   - 15.1 Description of mediators 37
   - 15.2 Results 39

16. Polygenic scores and occupational status trajectories throughout the careers 41

16.1 Data Preparation ____ 41
16.2 Analysis ____ 41
17. Polygenic score associations with health outcomes ____ 47
17.1 General health ____ 47
17.1a Controlling for parental occupational status ____ 48
17.1b Confounding of phenotypical effect of occupational status ____ 49
17.2 Mental health ____ 50
17.2a Controlling for parental occupational status ____ 52
17.2b Confounding of phenotypical effect of occupational status and mental health ____ 52
References ____ 55

List of Supplementary Figures

Supplementary Figure 1. Distribution of latest occupational prestige measured by ISEI from both the UK Biobank sample and the ONS sample

Supplementary Figure 2. Distribution of latest occupational prestige measured by SIOPS from both the UK Biobank sample and the ONS sample

Supplementary Figure 3. Phenotypic correlation (upper right triangle) versus Genetic correlation (lower left triangle) of occupational prestige and status measures

Supplementary Figure 4. Path diagram of Confirmatory Factor Analysis (CFA) for a general factor of occupational status

Supplementary Figure 5. Phenotypic correlation (upper right triangle) versus Genetic correlation (lower left triangle) of occupational status measures and other SES indicators

Supplementary Figure 6. Path diagram of Confirmatory Factor Analysis (CFA) for a general factor of socioeconomic status (using CAMSIS as the occupational indicator)

Supplementary Figure 7. Incremental R-square of polygenic score predictions of occupational status over the life course, NCDS

Supplementary Figure 8. Mediation results of polygenic prediction of occupational status (controlling for parental SES), NCDS

Supplementary Figure 9. Mean Percentile of the occupational status (CAMSIS) distribution across the career stratified by sex, parental education and the CAMSIS PGS. N = 201,939 time points from 5,475 individuals. Parental education measured as Low = No Qualifications, Medium = Lower Secondary, High = Upper Secondary/Degree.

Supplementary Figure 10. Mean Percentile of the occupational status (ISEI) distribution across the career stratified by sex, parental education and the ISEI PGS. N = 201,939 time points from 5,475 individuals. Parental education measured as Low = No Qualifications, Medium = Lower Secondary, High = Upper Secondary/Degree.

Supplementary Figure 11. Mean Percentile of the occupational status (SIOPS) distribution across the career stratified by sex, parental education and the SIOPS PGS. N = 201,939 time points from 5,475 individuals. Parental education measured as Low = No Qualifications, Medium = Lower Secondary, High = Upper Secondary/Degree.

Supplementary Figure 12. Mean Percentile of the occupational status (CAMSIS) distribution across the career stratified by career start and the CAMSIS PGS.

Supplementary Figure 13. Mean Percentile of the occupational status (ISEI) distribution across the career stratified by career start and the ISEI PGS.

Supplementary Figure 14. Mean Percentile of the occupational status (SIOPS) distribution across the career stratified by career start and the SIOPS PGS.

Supplementary Figure 15. Genetic confounding of correlations from respondent's occupational status to general health

Supplementary Figure 16. Genetic confounding of correlations from respondent's occupational status to mental health

List of Supplementary Tables

Supplementary Table 1. Results for BOLT-GREML SNP-heritability

Supplementary Table 2. Chi2 statistics for occupational status scores

Supplementary Table 3. Results of multivariate genetic regression models – CAMSIS and potential mediators

Supplementary Table 4. Results of multivariate genetic regression models – SIOPS and potential mediators

Supplementary Table 5. Results of multivariate genetic regression models – ISEI and potential mediators

Supplementary Table 6. Effect size reduction when controlling for parental SES

Supplementary Table 7. Spousal correlations of occupational status scores.

Supplementary Table 8. Spousal polygenic scores correlations of occupational status scores. PGS calculated with SBayesR weights.

Supplementary Table 9. Incremental R-square of polygenic score predictions of occupational status over the life course, NCDS (95% Cis). N = 5,389; 5,312; 5,211; 4,902; 4,263 for CAMSIS at age 33, 42, 46, 50 and 55, N = 5,449; 5,293; 5,197; 4,892; 4,252 for ISEI/SIOPS.

Supplementary Table 10. Variables used to construct general cognitive ability measure with corresponding loadings.

Supplementary Table 11. Variables used to construct scholastic motivation measure with corresponding loadings.

Supplementary Table 12. Variables used to construct externalizing behavior measure with corresponding loadings.

Supplementary Table 13. Variables used to construct internalising behavior measure with corresponding loadings.

Supplementary Table 14. Associations between occupational status PGS and general health at various ages, controlling for sex and first 10 PCs.

Supplementary Table 15. Associations between occupational status PGS and general health at various ages, controlling for father's occupational status at age 11, sex and first 10 PCs. Ratio denotes the ratio of the standardized beta-coefficient of the PGS without controlling for father's occupational status and the standardized beta-coefficient of the PGS when controlling for father's occupational status in the same sample of individuals for which paternal occupational status information was available.

Supplementary Table 16. Associations between occupational status PGS and mental health at various ages, controlling for sex and first 10 PCs.

Supplementary Table 17. Associations between occupational status PGS and mental health at various ages, controlling for father's occupational status at age 11, sex and first 10 PCs. Ratio denotes the ratio of the standardized beta-coefficient of the PGS without controlling for father's occupational status and the standardized beta-coefficient of the PGS when controlling for father's occupational status in the same sample of individuals for which paternal occupational status information was available.

1. Frequently Asked Questions (FAQs)

KEY-POINTS

This study examines **occupational status** (see **Glossary**) through a multidisciplinary lens to uncover the complex interplay between genetics and social environment.

The **primary method** to measure genetic associations with occupational status is a Genome-Wide Association Study (GWAS (see **Glossary**) in which a polygenic score is derived. From this, we construct polygenic scores and implement sibling family and adoption models to separate genetic and social environment associations. Crucially, the use of a GWAS is not an agenda to reduce occupational status to solely a genetic basis, but rather to examine the complex interplay between genetic and social environmental predictors and intergenerational transmission. Further multiple methods were used to uncover different aspects of this topic (see FAQ Figure 1).

We analysed data from **273,157 individuals** (130,952 males; 142,205 females) using the UK Biobank and **identified 106 independent genetic variant**s, including 8 newly associated with the genetics of socioeconomic status.

Key findings are:

- **Polygenic scores explains around 5-8% of the differences in occupational status amongst individuals**, more than three times as much as found in a previous study that used a cruder measure of occupation.
- Using a sibling research design – a technique that allows us to account for the shared family environment experience by siblings – the predictive power of these polygenic scores drops by over 50%. This suggests that **the polygenic score is also picking up non-genetic family and socio-environmental factors**, which we demonstrated previously for multiple complex behavioural phenotypes.
- As expected, **family environment remains an important factor in predicting occupational status of adults**. The family someone grows up in impacts an individual via what is termed gene-environment (GxE) correlation. We were able to empirically show this GxE correlation by examining parental occupational status from adoptees (i.e., children raised by non-biological parents) to those who were not adopted. Whereas the polygenic score dropped by over 50% when we compared siblings from the same family, the polygenic score's prediction of adoptees diminished by only roughly 25%.
- We show that this **54-57% reduction in predictability of polygenic scores within-families stems from strong socioeconomic status-based assortative mating (21-27%) over generations and indirect parental effects (22-27%)**. Indirect parental genetic effects are the influence of parental genotypes on their offspring over and above the transmission of genes, often described as genetic nurture. Parents transfer only around 50% of their own genetic material to their children and although parents may not have transmitted particular genetics to offspring, their own genetics may still impact their children indirectly through creating a family environment shaped by their own parental genetic makeup.
- The **intergenerational correlation of occupational status between parents and their children is only partly explained by genetic factors,** with **62% of the intergenerational correlation due to non-genetic factors** such as family environment and potentially rare

genetic variants. The rest is neither family nor genes but unique circumstances and factors that are still not measured. Notably, this is not specific to occupations, but to most complex diseases, behavioral, and social outcomes.

- Factors such as **cognitive skills, educational motivation, occupational aspiration, personality traits, and ADHD are the main drivers of the association between polygenic scores and occupational status**. Moreover, the **links between polygenic scores, career trajectories, and health are interrelated with parental occupational status**.
- We **examined the polygenic scores across 30-year career trajectories** to reveal that **societal structures correlate with genotypes and jointly predict career trajectories. Individuals who started in lower occupational status percentiles but ranked high in the PGS quintile for occupational status**, **consistency advanced their careers** over those 30 years. Those who held **higher occupational status jobs but had lower PGSs, exhibited a steady decline** in their professional trajectories.
- **There is a remarkably strong genetic correlation between genetic measures of other socioeconomic factors** (educational attainment and income). It is noteworthy that the genetic correlations observed among these socioeconomic indicators exceed the phenotypic correlations by a factor of two to three. Such a pattern is highly unusual in genomics and not observed for behavioral phenotypes or diseases.

Our findings illustrate the interdependence between genetic associations (measured by polygenic scores) and social environments (measured by familial/parental characteristics). Rather than nature versus nurture, we identify the importance of nature *and* nurture. Such a co-existence reiterates our previous claims and is further empirical demonstration that genetic results cannot and should not be used to in exclusion to predict an individual's occupational status or other complex behavioural outcomes.

WHY THIS FAQ?

This interdisciplinary study looks at occupational status and draws from multiple scientific approaches from the social sciences, molecular genetics, biostatistics and medical sciences. Given the controversial nature of examining differences in socioeconomic status and class in the context of the genome (see Box 1 main article) and potential of misinterpretation of our research, the aim of this FAQ is to create an accessible document for a broader audience to clarify what we conclude, and importantly, cannot conclude with this study. It is aimed at those who are new to the scientific terminology and methods (see **Glossary**). Experts or those seeking more in-depth information and scientific references supporting our statements, should refer to our main article and the detailed Supplementary Material.

WHAT DID WE STUDY?

The article by Akimova & Wolfram et al. (2024) examines **occupational status**, which is one of the core topics of social inequality and **social stratification research**, a field of research in the social sciences that studies and categorizes groups of people based on core socioeconomic (SES) factors like wealth, earnings, income, education, or occupation.

The majority of social stratification research has focused on the social determinants and social aspects of intergenerational transmission (i.e., from parent to offspring) of SES, often neglecting any role of biology or genetics. A growing number of studies found genetic variants linked to other SES indicators of education, income, and wealth. Yet to date, there was a lack of research examining occupational status, which is one of the most prominent measures of SES, particularly in disciplines such as sociology. We **used three measures** derived from decades of research in sociology, the: *International Socioeconomic Index (ISEI), Standard International Occupational Prestige Scale (SIOPS),* and *Cambridge Social Interaction and Stratification Scale (CAMSIS)*.

We therefore **performed a *GWAS* (Genome Wide Association Study) on measures of occupational status** along with various follow-up statistical analyses in order to understand the nature of discovered correlations and the **complex interplay between genes and environments**.

WHY STUDY THIS TOPIC NOW?

Socioeconomic status (SES) is a complex phenomenon influenced by behavior, biology, and the social environment. **Understanding SES requires a multidisciplinary approach** that identifies common drivers and addresses the multidimensional nature of their relationships with biology, health, social environments, and other behaviors.

Traditionally, biological and social processes of disease or complex behavior inheritance have been studied separately, often attributing them to social or biological factors and rarely considering both.

However, the joint consideration of social and biological factors in a biosocial model, provides a more comprehensive scientific understanding. Advances in technology, such as genotyping and large datasets that incorporate genetic and environmental/behavioral information, have spurred a wide range of empirical inquiries, enabling improved modeling of complex behaviors and *traits*.

Social stratification is a central predictor across the social and health sciences. The study of intergenerational status transmission and reproduction within families has received considerable attention in the literature. The focus has often been on how different family origins are associated with educational and labor market outcomes and the degree of occupational, class or social mobility from generation to generation. Here researchers often look at inequalities opportunities across countries and over time. Social science research has extensively demonstrated that socioeconomic status is influenced by multiple factors including sex and gender, family, societal and historical contexts, and social norms. Occupational status and mobility are shaped by inequalities of opportunity and also differ for men and women, often related to the opportunity costs and constraints of childbearing and rearing, but also when they were born and the geographical and policy context of individuals. Next to the family environment, parenting behavior and other investments in children, genetic inheritance plays a role.

For socioeconomic status measures such as educational attainment, nearly 4,000 *genetic variants* have been associated with the outcomes in previous studies. *Genetic variants* associated with income have also been assessed. Previous research has also shown that the third measure of occupational status, is not only driven by socio-environmental factors - twin studies suggest a *heritability* of occupational status as between 0.30 to 0.40. The current study goes substantially beyond what we know about the genetics and biological factors associated with occupational status.

Knowledge about potential genetic effects has led to various interpretations, often in relation to a measure of merit. Thus, a quantitative exploration of factors through which social and biological predictors are linked enhances our understanding of the nature of the links between the genome and social stratification in general. This approach helps prevent potentially misleading interpretations of latent genetic measures in the context of questions regarding equality of opportunity.

To date, the majority of research on differences in socioeconomic outcomes has been studied using a socially determinist approach, focusing only the role of social and contextual factors on prediction. More recently, however, researchers have conducted *genome-wide association studies (GWASs)*, which scans the entire genome to discover the genetics related to socioeconomic and other complex behavioral outcomes. Previous *GWASs* were conducted education, income, and wealth.

GLOSSARY

Assortative mating. Refers to a mating structure in which pairs of individuals that are (genetically) similar to each other mate with a higher probability than expected under random mating. Assortative mating is an important concept for statistical genetics; it biases heritability estimates.

Genetic ancestry. In the context of GWAS, genetic ancestry refers to the measure of genetic similarity among individuals to eliminate biases that are due to historical human migration. It should not be confused with race or ethnicity; it is also not a direct measure of genealogical ancestry.

Genetic associations. Refers to the relationship between single nucleotide polymorphisms (SNPs) and a particular outcome of interest. GWAS methodology tests for these associations across the genome. Significant associations identified in GWAS can then be used to create polygenic scores, where the effect sizes of selected SNPs are combined to predict the outcome.

Genetic variant. Refers to a specific region of the genome that differs between two genomes.

Genome-wide association study (GWAS). A GWAS is designed to adopt an hypothesis-free approach to discover genetic variants are associated with a trait. They often combine data from multiple studies to gather the largest sample possible. An updated and searchable list of all GWAS discoveries to date can be found at www.gwasdiversity.com, with summary statistics available at the GWAS Catalog.

Genotype. Describes part of an individual's DNA that influences their phenotype.

GWAS-heritability. The fraction of phenotypic variance of a trait explained by genome-wide significant genetic variants—sometimes also by polygenic scores based on GWAS findings.

Heritability. A population measure defining the proportion of variance in a phenotype explained by genetic variance within a population. We can differentiate between broad-sense heritability, including both additive and non-additive genetic effects such as epistasis and dominance, and narrow-sense heritability focusing on additive genetic effects only.

Indirect genetic effects. Refers to situations when environmental influences which are important for complex outcomes and phenotypes are also associated with individual's genotype. In such instances, environments referred as 'mediators' of the link between genetic variants and traits.

Occupational status. A measure developed primarily by sociologists to represent a stable indicator of an individual's social position in society. It is mapped g on a continuous scale by three measures. (1) International Socioeconomic Index (ISEI) (status measure constructed from scaling weights that maximize the (indirect) influence of education on income through occupation), (2) Standard International Occupational Prestige Scale (SIOPS), (prestige-based measure using public opinion surveys where a representative population is tasked with ranking occupations by their relative social standing), (3) Cambridge Social Interaction and Stratification Scale (CAMSIS) (measures distance between occupations based on the frequency of social interactions between them (operationalized as husband-and-wife combinations)).

Phenotype or trait. The observable characteristic of an individual, ranging from physical traits (hair colour, height) to disease status (diabetic) to behavior (risk-taker, age at first sexual intercourse, educational attainment).

Polygenic score (PGS). A single quantitative variable that summarizes genetic association to a phenotype by combining multiple genetic variants and their associated weights, derived from a GWAS. Polygenic scores for social outcomes are not tools to derive individual-level predictions but rather a population-level analytic tools.

Single-nucleotide polymorphism (SNP). A common variation in a single nucleotide (i.e., A, C, G, or T) that occurs at a specific position in the genome. A SNP exists as two different forms (e.g., A vs. T). These different forms are called alleles. A SNP with two alleles has three different genotypes (e.g., AA, AT, and TT).

SNP-heritability. The fraction of phenotypic variance of a trait explained by all SNPs in the analysis. Usually less than the narrow-sense heritability as it does not take rare variants and structural variation into account.

Why study occupation if we already know about education and income? Although they are related, having lower education does not always translate to a lower occupation or income. Conversely, someone might have high education, but not obtain a good job or high income. Others might have low income and rise high in the occupational prestige ranks. It is therefore interesting to understand whether there are genetic underpinnings of occupational status and how these genetics operate in relation to other socioeconomic outcomes, with health and across different environments, families and over time.

WHAT WAS THE AIM OF THIS STUDY?

The aim of this study is to improve our understanding of occupational status attainment and transmission, specifically through the complex interplay between biological inheritance and social processes.

To achieve this we:

- **Identified *genetic variants*** associated with our measures of occupational status to utilize them to **create polygenic scores**, which we used to control for genetic associations when studying factors related to socioeconomic status, occupations, labor market, and occupational mobility.
- **Examined the genetic correlates of occupation in relation to other socioeconomic indicators**.
- Investigated the **extent to which genetic associations of occupational status reflect the interplay between genetics, biology, family, social, and environmental factors**.
- Explored the **potential mechanisms linking the genome and occupational status**.
- Scrutinized the **underlying structure of the discovered associations**.
- Explored the **relationships between genes, occupational status, and (mental) health**.
- **Introduce a life course perspective** to examine how the polygenic scores operate over time as individuals age and progress through their careers.

HOW DID WE STUDY IT?

The **primary analysis we conducted is called a** ***Genome-Wide Association Study or GWAS*** (pronounced gee-was), which is a search across the entire human genome, examining each genetic locus (or region) one by one to see if there is a relationship (or what we call an association) between our outcomes and a particular *genetic variant*. *Variants* refer to a specific region of the genome, which differs between two genomes. Different versions of the same *variants* are termed alleles and a *SNP* (pronounced SNiP; *single-nucleotide polymorphism*) can have two alternative bases or alleles (C and T).

We study DNA *variants* that distinguish us from each other. Humans are 99.9% identical to each other, and it is the 0.1% by which we differ that makes us all genetically unique. A small subset of the 0.1% by which we differ genetically is anticipated to be associated with occupational status scores.

A comprehensive interdisciplinary study such as this one demanded multiple analytical approaches, we:

- **investigated the functional implications of *genetic variants*** associated with occupational status through gene-based and gene-set analyses using MAGMA technique.
- **employed multi-trait analysis (MTAG)** to meta-analyze occupational status measures with household income and educational attainment.
- **utilized genomic structural equation models (GSEM)** to analyze the joint factor of occupational status, cognitive performance, ADHD, openness to experience, risk tolerance, and neuroticism.
- engaged in ***polygenic score (PGS)* construction and prediction** involved producing various scores, testing out-of-sample prediction, and assessing *population stratification* using LD score regression.
- **applied sibling and adoption models** to disentangle direct, indirect, and demographic effects. To **study indirect 'social transmission' effects** we used two approaches: first, **adjusting our polygenic scores for parental SES** (measured by an individuals' parent's occupational status when they were aged 11. Second, **an adoption prediction study** to see if children raised by non-biological parents had different results.
- **conducted mediation and confounding analyses**.

HOW THE RESULTS SHOULD *NOT* BE INTERPRETED & WHAT ARE THE RISKS?

Are people biologically predetermined to have an occupational status?

No, that is not what we find. Our polygenic scores explain around 5-8% of the differences in occupational status amongst individuals but we also showed that when we account for the family environment (by looking at siblings), the predictive power of our polygenic scores drops by 50%, emphasizing the importance of family environment. But our results are still relevant since they show that the polygenic scores are picking up non-genetic family and social environment, which is relevant not only for our research but many complex outcomes. Importantly, we also provide empirical evidence that in addition to polygenic scores, family environment, socioeconomic based assortative mating over generations and the environment parents create (which is not only passing on genetics) are important. We also demonstrate that factors such as cognitive skills, educational motivation, occupational aspiration, personality traits, and ADHD are the main drivers of the association between polygenic scores and occupational status.

Could genetic results alone be used at the individual level to predict someone's occupational status?

No. That would be technically incorrect and a very bad idea. With the exception of some diseases, to date, polygenic scores alone are usually not useful to predict complex individual disease and behavioral outcomes. When we examine complex behavioral outcomes each individual *SNP* or *genetic variant* has a small effect, so prediction of using genetic results alone is not possible. Even if we combine the information contained in the more than 10 million *genetic variants* that we studied together into a genetic predictor, we predict 2-10% of the variance across individuals across different polygenic score approaches and occupational status measures. With larger samples we see that the ceiling of prediction is likely more in the range of 11-15% (depending on the measure of occupational status used). Extrapolating findings from other *traits*, more granular and detailed genetic data (on structural variation,

insertions, deletions and rare *variants*) might further increase this ceiling. For this reason, increasing standards are offered for using polygenic scores.

But it isn't a problem exclusive to genetics. Even the 'gold standard' social science predictors of occupational status, such as father's or mother's occupation or education when entered alone as a single variable in a regression equation would also low predictive power, generally under 10%. It is therefore unhelpfully reductive to think that it is useful or possible to enter one single variable as a predictor without considering additional factors. In reality, complex outcomes are a culmination of multiple factors such as genetics, parental background, lifestyle, level of education and national institutional configurations that constrain or enable behavior. We have even shown that the explanation of genetics can vary across country and time.

Should public officials, policy makers, insurers, or health care professionals use the polygenic score from this study to make decisions?
No. As noted above, genetics only predicts up to 10% of the variance in occupational status amongst individuals and is highly polygenic, meaning that it contains multiple genetic variants where we do not fully understand the biological causal function nor their interaction. As noted throughout, we empirically demonstrate the importance of family environment and socioeconomic factors and the interplay with genetics. The polygenic score alone should therefore not be used to make decisions.

Are there societal or medical implications of this study?
Analytical implications, most certainly. Societal maybe, but medical applications, extremely unlikely. In the longer term, this study offers a better understanding of the genetic architecture and responsible observable *traits* for occupational status. It equips scientists to take genetic effects into account in their study of SES and reduce bias due to genetic effects in their study of interest. But it also alerts medical and health researchers that polygenic scores for complex *phenotypes* are also picking up considerable social environmental and family effects and that *polygenic score* prediction vary by age.

We reveal changing genetic effects across the occupational career which opens an interesting puzzle for life course researchers with the potential for discoveries of lifestyle factors interacting with genes. Our analyses of the relationship between occupational status, genes and, (mental) health does have some **analytical ramifications for public health** as we can demonstrate that ignoring one of the dimensions produces a biased view on the other one. Furthermore, it is important to understand whether and which proportion of these *traits* are driven by genetic, behavioral and environmental factors. The fact that we also found evidence that genetic influences are much more shared than it's observed for different status measures suggests that continued research in this area is warranted to aid a better understanding of what makes the difference between income, education, and occupational status.

What are the potential risks of studying occupational status and genetics?
As outlined previously, the risks of introducing genomics in the study of occupation status for individuals are self-fatalism or self-stigmatization (i.e., believing their occupational status is fixed or inevitable or they are less capable). For society, the potential risks are discrimination against individuals (e.g., in employment, insurance, criminal justice), stigmatization of others or against entire groups, with potential for harmful or inequitably distributed policy

applications. Another risk is that genetics distracts from the real problem and channels resources away from more effective ways of addressing social stratification. Despite clear messaging, this research could also be misunderstood under the lens of genetic determinism and used to justify and reinforce existing inequalities as inevitable, hence seeing any interventions as futile. We recognize this apprehension and explicitly distance our research from studies that were (or are) overtly classist and/or racist and reinforce inequalities, confuse structural inequality with biology or draw overly-simplistic policy implications. Our endeavor, rather, is rooted in the pursuit of a biosocial understanding of occupational stratification, intergenerational transmission and the role that socio-economic status plays in genetic estimates, firmly guided by a well-established ethical, theoretical and analytical framework. Another risk is that superficial critics simply do not read the article and caveats we overtly present in the main article (Box 1) and here in the FAQ and incorrectly miscategorise our intentions and research.

WHO ARE WE AND WHO FUNDED THIS STUDY?

We are an interdisciplinary group of researchers working in the area of complex trait genomics, sociogenomics, statistical genetics and the social sciences. Although not exhaustive, we have published a broad variety of related work such as an MIT textbook on introducing quantitative statistical genetic data analysis (2021), identified statistical problems in genetic analyses (2021), the importance of country and historical time in genetic outcomes (2017) and problems with genetic essentialism narratives (2021). We have conducted previous GWASs on reproductive behavior, led by Mills (reproductive onset, age at first birth, number of children), published previously in *Nature Genetics* (2016), and *Nature Human Behaviour* (2021, 2023). We have also highlighted the importance of including family-data in GWAS research (*Nature Genetics*, 2022), lack of the data diversity and outcomes in GWAS discoveries (2019) and provide a daily update showing the lack of diversity in a GWAS Diversity Monitor (*Nature Genetics*, 2020). Mills has also published extensive non-genetic work in the social sciences on the topic of this outcome of occupational status and mobility in men and women and in relation to fertility (2012, 2021).

This project took multiple years and we are grateful for funding from various sources. Funding for this project for MCM and ETA is from the European Research Council ERC Advanced Grant CHRONO (835079), Leverhulme Trust (RC-2018-003) Leverhulme Centre for Demographic Science, and for MCM Economic and Social Research Council, United Kingdom Science and Innovation (UKRI) Connecting Generations Grant (ES/W002116/1), MapIneq Project, European Union's Horizon Europe research and innovation programme (No. 101061645) and for FCT UKRI FINDME (EP/Y023080/1) and AnalytiXIN, which is primarily funded through the Lilly Endowment, IU Health and Eli Lilly and Company.

WHAT ARE THE ETHICS APPROVAL OF THIS STUDY?

We are grateful for the contribution of all UK Biobank and NCDS participants to this scientific study. This research was conducted using the UK Biobank under application 32696 and NCDS under application GDAC_2021_16_TROPF, with ethical approval from the University of Oxford under application SOC_R2_001_C1A_21_60. Both the UK Biobank and NCDS applications were specific to the scope of this paper. For the UK Biobank approval, we received approval for a

scope extension to ensure transparency, allowing us to expand from our focus on non-standard occupations to also occupational status. Here was specified that our plan was: “to perform GWAS analysis using employment histories from the UK Biobank to construct sociologically informed measures of occupational status.” We specified that we would construct sociologically informed measures of occupational status (CAMSIS, SIOPS, and ISEA) for our GWAS and noted that the analysis would be accompanied by NCDS genetic and phenotypic data. For the NCDS application, we specified not only the information mentioned above but also the set of polygenic prediction analyses. We also preregistered our analysis plan (https://osf.io/djbr2/) which was updated for replication (https://osf.io/x6va5).

ADDITIONAL GENERAL QUESTIONS

Are the genetic associations small or large?

It is not really about size. Occupational status, similar to other socioeconomic measures, is a **complex outcome that is not only genetically based is largely predicted by social and family background factors** and a **complex interplay** with individual and socio-environmental contextual factors. As with any study that examines a complex behavioral outcome, genetics is only one piece of this larger puzzle. In this study we only examine common *genetic variants* (*SNPs*) and consider only one of the many possible biological and genetic ways in which individuals may vary. This does not impact the importance of the findings, since one single factor or variable ever fully explains complex outcomes. In earlier research, media and some scientists focused on the **overall ‘predictive power’**, which refers to the out of sample prediction of how much the genetic polygenic score alone predicts. In this study it is between 5-8%, depending on the occupational status and up to 9% depending on the career stage. But as noted throughout, scientists rarely ever use one predictor to explain an outcome, particularly a complex behavioral one like occupational status, BMI or Type 2 diabetes. It is always an interaction with multiple predictive factors.

Is it nature or nurture?

That is a **false dichotonomy** and it is neither nature or nature but rather nature *and* nurture. Occupational status – similar to other socioeconomic status measures or complex diseases - is a combination of both. Just as complex diseases such as obesity or Type 2 diabetes are neither purely genetically or socially determined, occupational status relates not only to biological factors, for example, influencing abilities or behavior, but also have a strong social and environmental component in that they are driven by one’s family, partner, job, and simultaneously shaped by the social, cultural, economic and historical environment. Genetic factors partly influence the first two factors of biological ability and behavior, complemented by social and environmental influences which also filter the types of behavior that are possible in the historical environment (e.g., via legislation, labor market structure, social norms).

What are the limitations of this study?

Although we open up new avenues of research, there are limitations that are not exhaustive or exclusive to this type of study, with the central ones are listed here. First, we focus on British-European genetic ancestry individuals only, a problem we have highlighted elsewhere. We conducted a scientometric review of all *GWAS* and found that 72% of genetic discoveries come from 3 countries, and therefore set up the GWASDiversityMonitor described in our Nature

Genetics article. Second, we draw most of our results from the UK Biobank, which is a selective population that has fewer health problems and a higher SES. Such a participation bias limits the generalizability and introduces the potential that observed genetic associations may be influenced by the characteristics of the subset of individuals who chose to participate in the UK Biobank. A recent *Nature Genetics* (2023) study by colleagues working in our own Leverhulme Centre for Demographic Science (LCDS) explain why this is an issue for genetic research, also described here. Third, due to data limitations, we recognize that we were unable to also include parent's polygenic scores to estimate genetic confounding effects, which we have shown can be problematic (*Nature Genetics*, 2022), as have our own LCDS researchers in our centre in other publications (*Science*, 2018).

Data availability

The GWAS summary statistics generated in this study are available on the GWAS Catalog website under accession codes GCST90446160, GCST90446162, GCST90446163. Access to the UK Biobank is available through: http://www.ukbiobank.ac.uk). Access to The National Child Development Study (NCDS) is available through: https://cls.ucl.ac.uk/data-access-training/.

References

Akimova, E. T., Breen, R., Brazel, D. M., & Mills, M. C. (2021). Gene-environment dependencies lead to collider bias in models with polygenic scores. *Scientific Reports*, *11*(1), 9457.

Barban, N., Jansen, R., De Vlaming, R., Vaez, A., Mandemakers, J. J., Tropf, F. C., ... & Hopper, J. (2016). Genome-wide analysis identifies 12 loci influencing human reproductive behavior. *Nature genetics*, *48*(12), 1462-1472.

Barth, D., Papageorge, N. W., & Thom, K. (2020). Genetic endowments and wealth inequality. *Journal of Political Economy*, *128*(4), 1474-1522.

Begall, K., & Mills, M. C. (2013). The influence of educational field, occupation, and occupational sex segregation on fertility in the Netherlands. *European sociological review*, *29*(4), 720-742.

Benjamin, D. J., Cesarini, D., Chabris, C. F., Glaeser, E. L., Laibson, D. I., Age, Gene/Environment Susceptibility-Reykjavik Study:, ... & Lichtenstein, P. (2012). The promises and pitfalls of genoeconomics. *Annu. Rev. Econ.*, *4*(1), 627-662.

Benonisdottir, S., & Kong, A. (2023). Studying the genetics of participation using footprints left on the ascertained genotypes. *Nature Genetics*, *55*(8), 1413-1420.

Berryessa, C. M., & Cho, M. K. (2013). Ethical, legal, social, and policy implications of behavioral genetics. *Annual Review of Genomics and Human Genetics*, *14*(1), 515-534.

Blossfeld, H. P., Mills, M., & Bernardi, F. (Eds.). (2006). *Globalization, uncertainty, and men's careers: an international comparison*. Edward Elgar Publishing.

Breen, R., & Jonsson, J. O. (2005). Inequality of opportunity in comparative perspective: Recent research on educational attainment and social mobility. *Annu. Rev. Sociol.*, *31*(1), 223-243.

Briley, D. A., Harden, K. P., & Tucker-Drob, E. M. (2014). Child characteristics and parental educational expectations: Evidence for transmission with transaction. *Developmental psychology*, *50*(12), 2614.

de Hemptinne, M. C., & Posthuma, D. (2023). Addressing the ethical and societal challenges posed by genome-wide association studies of behavioral and brain-related traits. *Nature neuroscience*, *26*(6), 932-941.

Engzell, P., & Tropf, F. C. (2019). Heritability of education rises with intergenerational mobility. *Proceedings of the National Academy of Sciences*, *116*(51), 25386-25388.

Herd, P., Mills, M. C., & Dowd, J. B. (2021). Reconstructing sociogenomics research: Dismantling biological race and genetic essentialism narratives. *Journal of health and social behavior*, *62*(3), 419-435.

Hill, W. D., Davies, N. M., Ritchie, S. J., Skene, N. G., Bryois, J., Bell, S., ... & Deary, I. J. (2019). Genome-wide analysis identifies molecular systems and 149 genetic loci associated with income. *Nature communications*, *10*(1), 5741.

Hingorani, A. D., Gratton, J., Finan, C., Schmidt, A. F., Patel, R., Sofat, R., ... & Wald, N. J. (2023). Performance of polygenic risk scores in screening, prediction, and risk stratification: secondary analysis of data in the Polygenic Score Catalog. *BMJ medicine*, *2*(1).

Howe, L. J., Nivard, M. G., Morris, T. T., Hansen, A. F., Rasheed, H., Cho, Y., ... & Davies, N. M. (2022). Within-sibship genome-wide association analyses decrease bias in estimates of direct genetic effects. *Nature genetics*, *54*(5), 581-592.

Ko, H., Kim, S., Kim, K., Jung, S. H., Shim, I., Cha, S., ... & Won, H. H. (2022). Genome-wide association study of occupational attainment as a proxy for cognitive reserve. *Brain*, *145*(4), 1436-1448.

Kong, A., Thorleifsson, G., Frigge, M. L., Vilhjalmsson, B. J., Young, A. I., Thorgeirsson, T. E., ... & Stefansson, K. (2018). The nature of nurture: Effects of parental genotypes. *Science*, *359*(6374), 424-428.

Marmot, M. (2015). The health gap: the challenge of an unequal world. *The Lancet*, *386*(10011), 2442-2444.

Mathieson, I., Day, F. R., Barban, N., Tropf, F. C., Brazel, D. M., eQTLGen Consortium, ... & Perry, J. R. (2023). Genome-wide analysis identifies genetic effects on reproductive success and ongoing natural selection at the FADS locus. *Nature human behaviour*, *7*(5), 790-801.

Meyer, M. N., Appelbaum, P. S., Benjamin, D. J., Callier, S. L., Comfort, N., Conley, D., ... & Parens, E. (2023). Wrestling with social and behavioral genomics: risks, potential benefits, and ethical responsibility. *Hastings Center Report*, *53*, S2-S49.

Mills, M. C., Barban, N., & Tropf, F. C. (2020). *An introduction to statistical genetic data analysis*. Mit Press.

Mills, M. C., & Rahal, C. (2019). A scientometric review of genome-wide association studies. *Communications biology*, *2*(1), 1-11.

Mills, M. C., & Rahal, C. (2020). The GWAS Diversity Monitor tracks diversity by disease in real time. *Nature genetics*, *52*(3), 242-243.

Mills, M. C., Tropf, F. C., Brazel, D. M., van Zuydam, N., Vaez, A., Agbessi, M., ... & Human Reproductive Behaviour Consortium. (2021). Identification of 371 genetic variants for age at first sex and birth linked to externalising behaviour. *Nature human behaviour*, *5*(12), 1717-1730.

Okbay, A., Wu, Y., Wang, N., Jayashankar, H., Bennett, M., Nehzati, S. M., ... & Young, A. I. (2022). Polygenic prediction of educational attainment within and between families from genome-wide association analyses in 3 million individuals. *Nature genetics*, *54*(4), 437-449.

Tambs, K., Sundet, J. M., Magnus, P., & Berg, K. (1989). Genetic and environmental contributions to the covariance between occupational status, educational attainment, and IQ: A study of twins. *Behavior genetics*, *19*, 209-222.

Treiman, D. J. (2013). *Occupational prestige in comparative perspective*. Elsevier.

Tropf, F. C., Lee, S. H., Verweij, R. M., Stulp, G., Van Der Most, P. J., De Vlaming, R., ... & Mills, M. C. (2017). Hidden heritability due to heterogeneity across seven populations. *Nature human behaviour*, *1*(10), 757-765.

Verweij, R. M., Stulp, G., Snieder, H., & Mills, M. C. (2021). Explaining the associations of education and occupation with childlessness: the role of desires and expectations to remain childless. *Population Review*, *60*(2), 166-194.
Wand, H., Lambert, S. A., Tamburro, C., Iacocca, M. A., O'Sullivan, J. W., Sillari, C., ... & Wojcik, G. L. (2021). Improving reporting standards for polygenic scores in risk prediction st

2. Background

Occupational status – including its prestige and other sociological factors - is a crucial component of socioeconomic status (SES) and correlates with many physical and mental illnesses as well as longevity.[1,2] Explanations for the SES-health gradient have also been examined in the context of genetics. Recent Genome-Wide Association Studies (GWASs) have identified the genetic contributions to economic status for education[3-5] and income.[6,7] Research on the genetic basis of occupation has to date focused mainly on entrepreneurship[8] and occupation-related stress and exhaustion.[9,10] Little is known about the genetic associations with occupational status and prestige, which is related to an individual's education and income level, and signals their social standing.[11]

The current study is a GWAS of sociologically informed measures of occupation, extending previous work in several ways. Our analyses focus on the International Socioeconomic Index (ISEI), the Standard International Occupational Prestige Scale (SIOPS), and the Cambridge Social Interaction and Stratification Scale (CAMSIS). In the following we detail our analyses which had been preregistered February 2nd, 2021 (https://osf.io/djbr2/) and updated for replication (https://osf.io/x6va5).

3. Measuring occupational status and prestige

The three approaches from the sociological tradition to measuring occupational status and prestige either consider socioeconomic differences between occupations, interoccupational social interaction or ascribed prestige of different jobs.[12]

Dating back to their earliest inception in the 1950s[13,14] *socioeconomic difference*-based indices measure the "attributes of occupations that convert a person's main resource (education) into a person's main reward (income)".[15] The most used measure of this tradition is the *International Socioeconomic Index* (ISEI[15]), which is a measure of prestige or status constructed from scaling weights that maximize the (indirect) influence of education on income through occupation.

More focused *prestige*-based measures on the other hand are simply the result of public opinion surveys in which representative samples of the population are tasked with ranking occupations by their relative social standing, emerging at a similar time as socioeconomic difference-based indices (e.g., Nakao and Treas 1992[16]). Treiman (1977)[17] showed in an extensive analysis that prestige-based measures were surprisingly constant over time and cultures, cementing their use in social scientific research. His *Standard International Occupational Prestige Scale* (SIOPS or Treiman-prestige[18]) remains one of the most commonly used metric in this tradition.

Lastly, occupational status indicators derived from *social interaction* focus on the heterogeneity of associations between occupants of different jobs, following the tradition of Warner, Meeker, and Eells (1949)[19] and Laumann and Guttman (1966).[20] They are based on the idea that differential association is a function of social stratification, as members of a group are more likely to interact with members of that group than with members of other groups. Thus, acquaintances, friends, and spouses are much more likely to be selected from within the

same group than an outgroup. A group of Cambridge sociologists reversed this approach to measure occupational structure based on interactions. The *Cambridge Social Interaction and Stratification Scale* (CAMSIS) measures the distance between occupations based on the frequency of social interactions (operationalized as husband-and-wife combinations) between them.[21]

4. Research plan

Analyses followed the pre-registration uploaded by Brazel, Ding and Mills to the open science framework on February 2nd, 2021 and updated in February 2023 also including CAMSIS (https://osf.io/x6va5).

The focus was on the most recent occupation the participant held. We studied ISEI, SIOPS, CAMSIS, the (within-family) prediction of polygenic signals and beyond. Analysts (ETA, TW) achieved genetic correlations of 1 in their exploration.

5. Phenotype definitions

Discovery is conducted in the UK Biobank, which is a large prospective cohort study in the United Kingdom (UK), following over 500,000 volunteers who were between 40 and 69 years of age at the time of their recruitment between 2006 and 2010.[22,23] The definition used by researchers conducting the analysis is now outlined below.

Current or most recent occupation is treated as a continuous measure. UK Biobank respondents were asked to provide job titles for the current or the most recent job held. The job information was coded using the four-digit UK Standard Occupational Code version 2000 (SOC2000). We built a procedure to link the UK SOC2000 to ISCO-88(COM), and then derive ISEI and SIOPS from ISCO-88(COM). All phenotypes are inverse-normal rank transformed before analysis. The CAMSIS-based status could be directly merged using the data available from Lambert and Prandy (2018).[24] ISEI and SIOPS (as provided by the R-package "strat"[25]), however, use the less granular ISCO-88[26] scale, so a mapping from ISCO to SOC2000[27] was employed. If multiple job codes for a respondent were available, the newest was used.

For some of our analyses, we rely on the genotyped subsample of the National Child Development Study (NCDS). Here, the SOC2000 code of the respondent's occupation (as well as their father's when they were 11 years old), is available as well, so a similar procedure is applied.

We also measured other SES dimensions such as income as done by Hill et al. (2019)[7] using a coarse, 5-level ordinal household income variable. Educational attainment was defined as years of education and coded according to the scheme provided by Lee et al. (2018).[3]

6. Representativity of the UK Biobank with the Office of National Statistics (ONS)

Supplementary Figure 1 and Supplementary Figure 2 show the distribution of latest occupational status measured by ISEI and SIOPS from both the UK Biobank sample and the Office of National Statistics (ONS) sample. The purpose of comparing the distribution of the two samples is to check whether our phenotypic distribution of occupational status is broadly representative of the national population. It is categorized into eight (ISEI) and seven (SIOPS) groups.

We also compare the sex ratio of each occupational group in the two samples. Supplementary Figure 1 and Supplementary Figure 2 demonstrate that the UK Biobank sample is largely in line with the general population. However, we found that the UK Biobank sample in general under-represents individuals in the lower end of the occupational prestige and over-represents those in the higher end of the occupational prestige. In addition to potential sample selectivity of the UK Biobank, this is also attributed to the fact that the UK Biobank sample covers individuals over 40 years old whereas the ONS data source samples the entire labor force. The sex ratio of the UK Biobank and UK population sample are highly correlated.

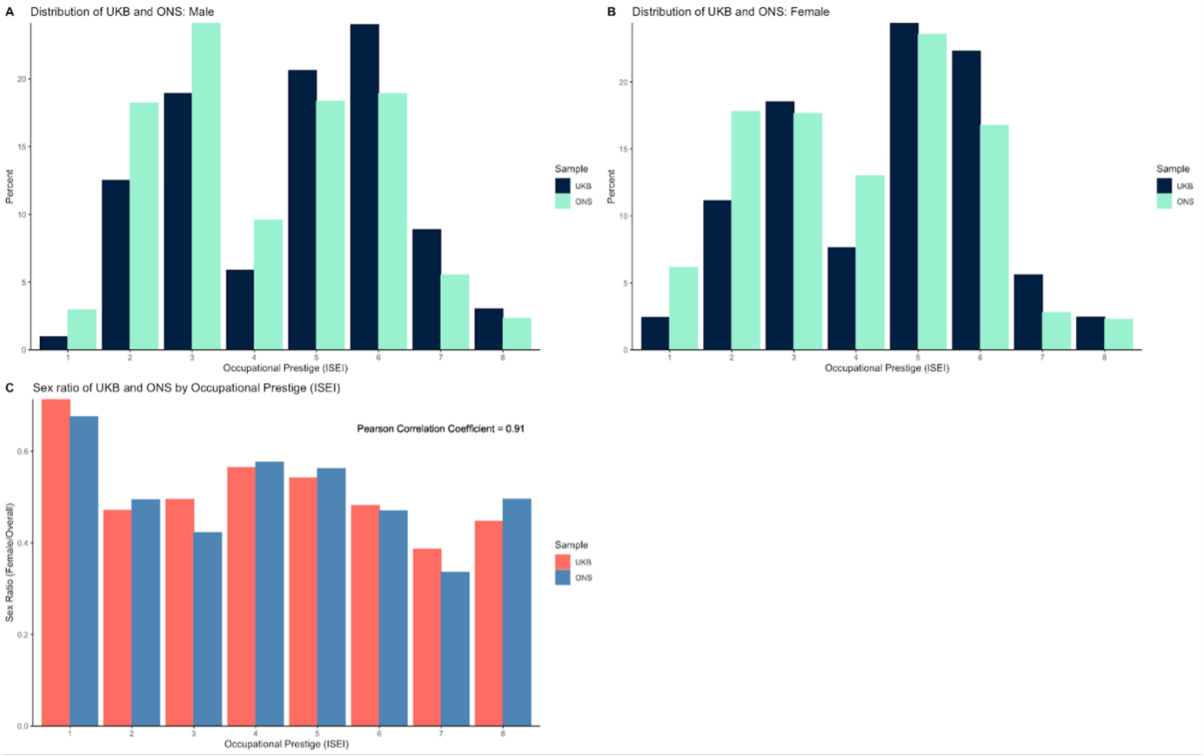

Supplementary Figure 1. Distribution of latest occupational prestige measured by ISEI from both the UK Biobank sample and the ONS sample

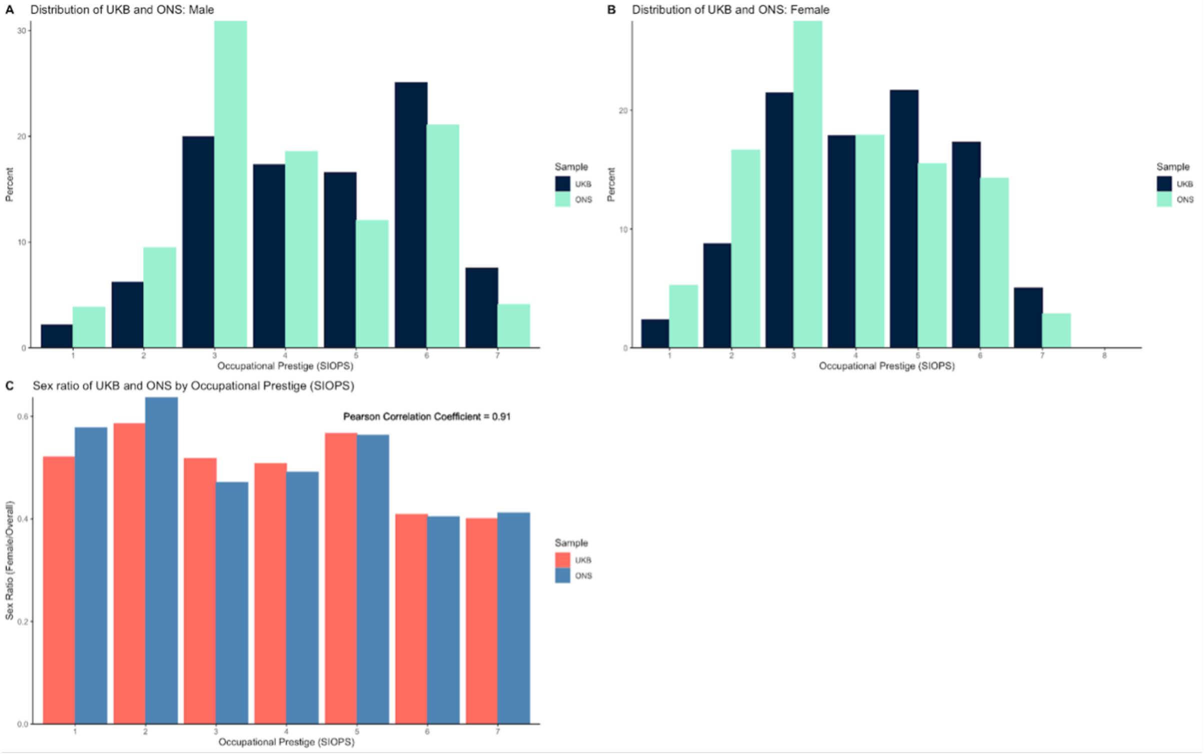

Supplementary Figure 2. Distribution of latest occupational prestige measured by SIOPS from both the UK Biobank sample and the ONS sample

7. Overview of GWAS analyses

For discovery, we followed the analysis plan first uploaded by Brazel, Ding, and Mills to the open science framework on February 2nd, 2021, which was updated including CAMSIS (https://osf.io/djbr2/) in February 2023 (https://osf.io/x6va5).

7.1 Analyses

All calculations are based on mixed model association tests as implemented in the program FastGWA,[28] with association testing based on v3 imputed data. Following the pre-posted open science analysis plan in each regression, the following covariates are included: The first 10 genomic principal components, age at assessment and age^2, UK Biobank assessment center at recruitment, sex, genotyping array (BiLEVE or Axiom) on the sample of British-European genetic ancestry. Genetic ancestry was determined based on principal components (PC) analysis of the genetic data. Chromosomes are analyzed separately. To speed up the calculation of summary statistics, a minimum MAF filter of 0.01 was imposed, leaving 10.2 million SNPs for the analysis.

7.2 Sample inclusion criteria

Individuals were included from British-European genetic ancestry who had information for the phenotype in question, all relevant covariates, no mismatch between submitted and inferred

gender, no outlier for heterozygosity and missingness, no evidence for sex chromosome aneuploidy and if they were present in all relevant genetic datasets (unimputed, imputed autosomal and imputed X chromosome), leaving in total 273,157 (130,952 males, 142,205 females) and 271,769, (130129 males, 141640 females) individuals for the occupational status phenotypes (CAMSIS and SIOPS/ISEI) and 353,673 (1692,01 males, 184,472 females) and 404,420 (185,632 males, 218,788 females) individuals for the secondary analyses (household income and education), respectively. For all analyses based on the sibling sample of the UK Biobank, separate GWAS using the same specifications with the respective individuals removed were conducted. We focus on individuals of British-European genetic ancestry only in order to decrease the risk of confounding due to population stratification.

7.3 Findings

After inflating the standard errors by the square root of their respective intercepts from LD Score regressions, our GWASs identified 106 independent SNPs for CAMSIS including 56 found also for ISEI and 51 for SIOPS based on clumping all genome-wide significant SNPs using a threshold of 0.1 and a window-size of 1000kb, one of which (only significant for CAMSIS) was found on the X-chromosome (see Figure 2 in the main text for the Manhattan plot of the autosome).[1] In exploratory sex-specific GWAS, no separate hits emerged, with genetic correlations being very close to and not significantly different from one.

7.4 Replication

To validate our findings, we replicated our top hits using the genotyped subsample of the National Child Development Study (NCDS), an ongoing British Birth Cohort Study which started in 1958 and includes ~6500 individuals with both genetic and phenotypical information.

We replicate our findings on CAMSIS, which includes the discovery for ISEI and SIOPS, exhibited the highest SNP-heritability (see SI 7) and achieved the best polygenic prediction (SI 10), showing a genetic correlation not significantly different from 1 to ISEI and SIOPS (SI 11). To keep in line with the age range of the discovery sample, we used occupational information later in life (age 50). After filtering for standard quality control measures like those in the discovery, the final dataset included 4,899 individuals with 10 PCs and sex as covariates.

Restricting our pre-clumped summary statistics to SNPs found in the NCDS, we were able to match 103 of the 105 autosomal independent genome-wide significant variants (or variants in LD to them) between both datasets for replication.

Considering the limitations imposed by the smaller sample size of the NCDS dataset, we turned to the methods presented by Okbay, Beauchamp, Fontana, Lee, Pers, Rietveld et al. (2016, SI 1.8.3),[29] to assess the expected sign concordance and significance of our results, allowing us to estimate the expected performance of our replication while considering the standard errors in both discovery and replication datasets.

The probability that the SNPs have the same sign in both discovery and replication datasets, $P(\text{match})$, is:

[1] We are using PLINK --clump command with the specifications we described above.

$$P(\text{match}) = \Phi\left(\frac{-|\beta|}{\sigma_{\text{GWAS}}}\right)\Phi\left(\frac{-|\beta|}{\sigma_{\text{replication}}}\right) + \left(1-\Phi\left(\frac{-|\beta|}{\sigma_{\text{GWAS}}}\right)\right)\left(1-\Phi\left(\frac{-|\beta|}{\sigma_{\text{replication}}}\right)\right)$$

where β is the vector of winners curse corrected effect sizes froms the discovery, σ_{GWAS} the vector of associated standard errors in the discovery and $\sigma_{\text{replication}}$ the vector of standard errors in the replication. Then $\Sigma P(\text{match})$ is the expected number sign-concordant SNPs.

The NCDS replication significantly outperformed expectations under the null hypothesis that discovered signals are not robust. At $p < 5 \times 10^{-8}$, the expected sign concordance was 54.2 under the null while the actual match was 67. At the suggestive significance level of $p < 5 \times 10^{-6}$, the expected sign concordance was 199.2, and the actual match was 255.

The probability of significant hits on a given α-level, $P(\text{sig})$, can be computed as:

$$P(\text{sig}) = \Phi\left(\frac{-|\beta|}{\sigma_{\text{replication}}} + \Phi^{-1}\left(\frac{\alpha}{2}\right)\right) + \left(1-\Phi\left(\frac{-|\beta|}{\sigma_{\text{replication}}} - \Phi^{-1}\left(\frac{\alpha}{2}\right)\right)\right)$$

Again, we can sum over the vector $\Sigma P(\text{sig})$ to get the expected number of hits.

Demonstrating the consistency of our findings, the number of significant hits at α = 0.05 was in line with expectations: At $p < 5 \times 10^{-8}$, the expected match was 5.2, and the actual match was 5 and at $p < 5 \times 10^{-6}$, an actual match of 24 was achieved, compared to an expectation of 19.4.

8. SNP-heritability

SNP-heritability for all three primary (ISEI, SIOPS, CAMSIS) and the two related SES phenotypes (education, household income - analyzed in the UK Biobank) were computed using LDSC[30] from GWAS summary statistics. All measures exhibit SNP-heritability significantly larger than zero, as shown in Figure 3 in the main text. The values for household income and educational attainment are comparable to SNP-heritability previously published in the literature.[3,7] Heritability of ISEI and SIOPS is 0.10 to 0.11 and larger in CAMSIS, comparable to that of educational attainment, with 0.146.

In addition and as a robustness check, SNP-heritability was also estimated using BOLT-GREML on the GWAS samples. The results confirm and even exceed the LDSC estimates.

Outcome	SNP-h2 (GREML)	SE
CAMSIS	0.152535	0.002779
ISEI	0.119530	0.002696
SIOPS	0.114053	0.002680
Years of Education	0.162157	0.002790
Household Income	0.094426	0.002843

Supplementary Table 1. Results for BOLT-GREML SNP-heritability

9. Population stratification test

We applied LD Score intercept method[31] to assess whether population stratification influenced our findings or potentially resulted in false positives. In doing so, we used the LDSC software[32] to calculate LD Score regressions for each occupational score separately. LD Scores were computed utilizing genotypic data from individuals of European genetic ancestry within the 1000 Genomes Project, specifically focusing on HapMap3 SNPs. Inclusion in the LD Score regression analysis was limited to HapMap3 SNPs with a minor allele frequency (MAF) exceeding 0.01 only.

Supplementary Table 2 below demonstrates the results of our analyses. It is noticeable that LD Score intercepts deviate statistically significantly from 1, although the deviation is not substantial. The chi^2 statistics fall within the range of 1.53 to 1.74 for each occupational score. We thus find evidence supporting the notion that a portion of the identified SNPs are linked to our phenotypes, and approximately 15.8% to 17.5% of the observed inflation in chi^2 can be attributed to potential biases arising from factors such as population stratification and other confounders. Hence, our overall findings indicate evidence that the predominant portion of the observed signal is attributable to polygenic factors but the biases due to stratification or relatedness explain on average 16.5% of the inflation of chi^2.

Outcome	Mean chi^2	Intercept (SE)	Inflation
CAMSIS	1.738	1.1193 (0.0142)	0.1616531
ISEI	1.568	1.0993 (0.0134)	0.1748239
SIOPS	1.534	1.0845 (0.0123)	0.1582397

Supplementary Table 2. Chi^2 statistics for occupational status scores

10. Polygenic score calculation and prediction

10.1 Calculation of polygenic scores

Overall, we calculated three types of polygenic scores for each phenotype:

1. **Pruning and thresholding polygenic scores using PRSice.**[33]
   Polygenic scores were calculated using a prespecified threshold of p = 0.5 in the sample and with the software default values for clumping (250kb window; r^2=.1).
2. **SBayesR[34] polygenic scores.** Polygenic scores were calculated using the software default values (pi = 0.95,0.02,0.02,0.01, gamma = 0,0.01,0.1,1) and a shrunk sparse LD-matrix computed based on 1.1 million common SNPs in a random sample of 50K unrelated individuals of British-European genetic ancestry in UK Biobank provided at https://zenodo.org/record/3350914#.XyFfnC17G8o.[34]
3. **MTAG+ SBayesR polygenic scores.** We calculated this type of polygenic scores using the same specifications as in SBayesR polygenic scores but instead it is based on MTAG results[35] from GWAS on all occupation scores[2] and the secondary GWAS on income and education. To maximize our predictive power, in the NCDS the EA3 GWAS[3] (excluding NCDS) is used instead of the secondary UKB education GWAS.

10.2 Out-of-sample prediction

For all phenotypes, we use OLS regression models and report the incremental R-squared over a baseline model consisting of 10 principal components, sex and age. Accordingly, we investigated the predictivity of polygenic scores in the subsample of siblings in the UK Biobank (N = 24,579 for CAMSIS and 24,472 for ISEI and SIOPS, we ran additional GWAS excluding these observations from the sample) and in the genotyped subsample of the National Child Development Study (NCDS), an ongoing British Birth Cohort Study started in 1958. For the NCDS, observations for current occupations were pooled over all waves starting at age 33 (N = 5,389; 5,312; 5,211; 4,902; 4,263 for CAMSIS at age 33, 42, 46, 50, and 55, N = 5,449; 5,293; 5,197; 4,892; 4,252 for ISEI/SIOPS). Figure 4 in the main text demonstrates the results of performance of different polygenic scores.

11. Uncovering genetic communality of occupational status and prestige with socio-economic and other measures using Genomic SEM

11.1 General factor of occupational status

[2] The use of all occupational status scores GWAS for MTAG also has a theoretical and substantive motivation. While ISEI, SIOPS, and CAMSIS have genetic correlations > 0.95 (Supplementary Figure 3), each of these occupational measures captures slightly different dimensions of occupational status. Accordingly, we use MTAG to take into account a broader spectrum of occupational characteristics and to ensure that we do not overlook subtle, yet potentially meaningful nuances within each measure.

We observed strong correlations between all three occupational prestige and status measures. Supplementary Figure 3 below illustrates an extremely high genetic correlation between CAMSIS, ISEI, and SIOPS (lower triangle), much stronger than implied by their phenotypic correlations (upper triangle). We also observe a substantial genetic correlation with the SOC2000 index used by Ko et. al.[36], while phenotypic correlations are of a lesser magnitude.[3]

Using genomic structural equation modelling (R package GenomicSEM)[37] on the genetic correlation matrix, we find clear evidence for a general factor of occupational status (Supplementary Figure 4) with extremely high loadings of all three measures. It is much higher than a phenotypic exploratory factor analysis in the same sample (path coefficients of 0.86, 0.96 and 0.94, for CAMSIS, ISEI and SIOPS, respectively, not shown).

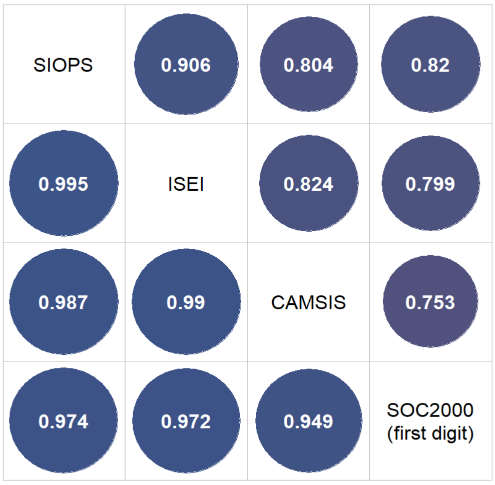

Supplementary Figure 3. Phenotypic correlation (upper right triangle) versus Genetic correlation (lower left triangle) of occupational prestige and status measures. Darker blue circles indicate stronger positive correlations.

[3] We would like to point out that although our genetic correlations with the measures previously used by Ko et al. are high, our SNP heritability estimates are almost twice as high, especially for CAMSIS. This similarity in genetic correlations, despite the substantial difference in LDSC-based SNP heritability, is somewhat expected. These are independent measures, and high genetic correlations can occur between outcomes independent of heritability. Moreover, we also expect variation in validity among different occupational scores (i.e., different measures capture the intended concepts to varying extents), which likely plays a role in the observed high genetic correlations and substantial differences in SNP heritabilities.

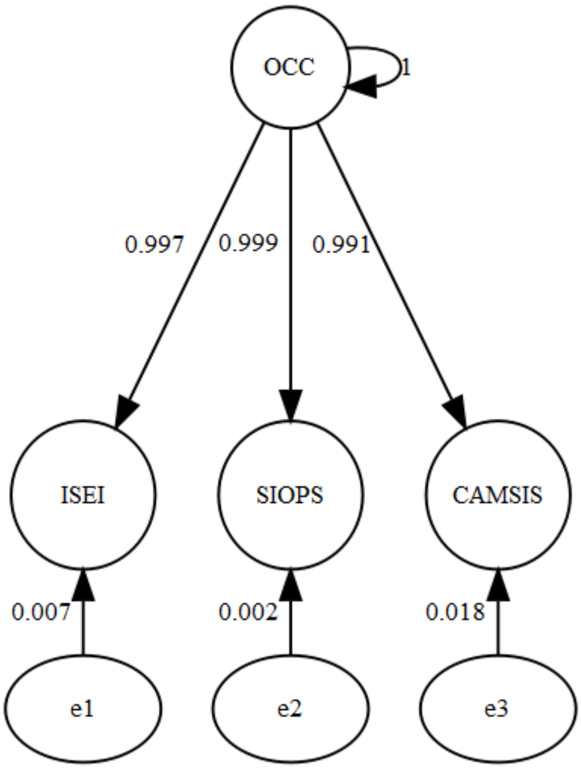

Supplementary Figure 4. Path diagram of Confirmatory Factor Analysis (CFA) for a general factor of occupational status

11.2 General factor of socioeconomic status (SES)

In addition to exhibiting strong genetic commonality, Supplementary Figure 5 shows that genetic correlations of CAMSIS, ISEI and SIOPS with educational attainment and household income (lower triangle) are without exception in the extreme as well. This is in particular evident in contrast to the phenotypic correlations (upper triangle), which are existent and positive, but much smaller.

This implies the existence of a common factor of socioeconomic status, consisting of occupation, education, and income. This is again supported by factor analysis using genomic SEM (Supplementary Figure 6).

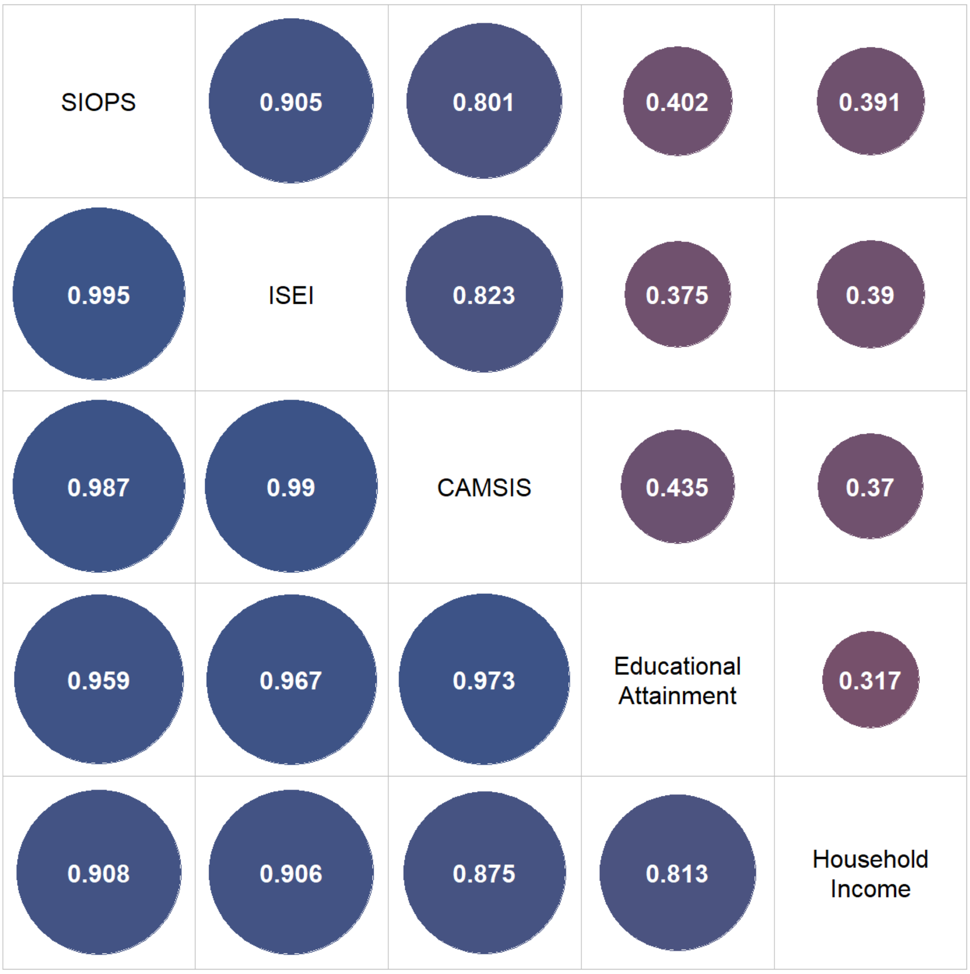

Supplementary Figure 5. Phenotypic correlation (upper right triangle) versus Genetic correlation (lower left triangle) of occupational status measures and other SES indicators. Darker blue circles indicate stronger positive correlations.

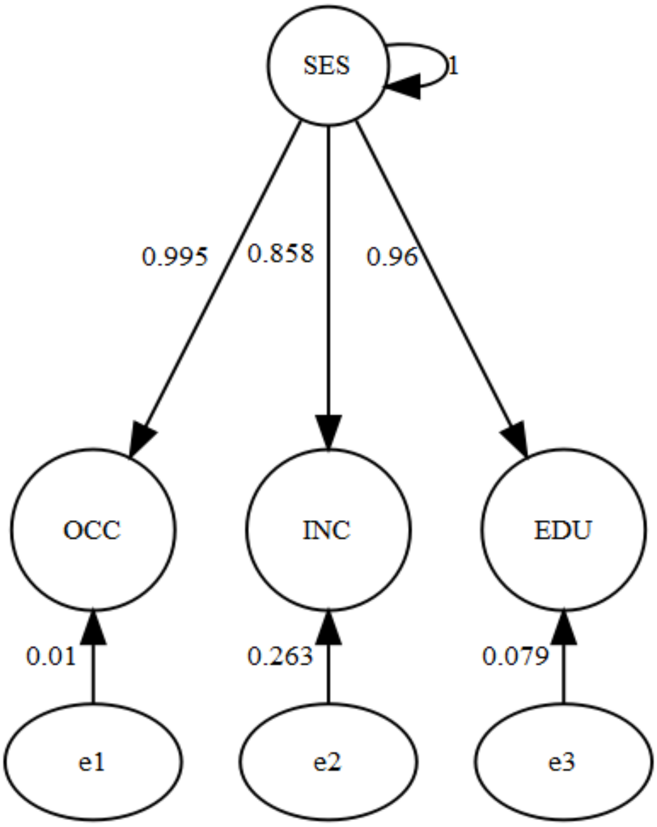

Supplementary Figure 6. Path diagram of Confirmatory Factor Analysis (CFA) for a general factor of socioeconomic status (using CAMSIS as the occupational indicator)

11.3 Mediators between polygenic signals and occupational status

What biologically proximal traits are responsible for the common factor underlying genetic variation in socioeconomic status? Evidence from twin studies points to the direction of cognitive and noncognitive traits acting as mediators,[38] showing for example that the heritability of education is mostly due to a variety of noncognitive factors and intelligence,[39] all of which show substantial genetic influence.[40-43]

We validate these findings by identifying five causally upstream traits from the genetic literature that we expect to mediate the general genetic factor of socioeconomic status, namely cognitive performance,[3] ADHD (as a proxy for behavioural disinhibition),[44] openness to experience,[45] risk tolerance,[46] and neuroticism[47] and fitting a multivariate genetic regression model[48] in Genomic SEM.

Results presented in Supplementary Tabkes 3-5 show the respective regression results, which indicate that overall, almost 70% of the heritability in the three occupational status measures captured in our GWASs can be explained by genetic correlations with GWAS summary statistics

of just these five traits. Between traits, associations are largely similar: The strongest effects are observable for cognitive performance. However, they are reduced by more than 30% once ADHD and particularly openness are added to the model. The influence of ADHD is then itself increased by the introduction of risk tolerance, whose effect on the SES factor is – in contrast to ADHD and neuroticism – positive. The latter is furthermore the only covariate with heterogeneous effects. While significantly negatively associated with SIOPS and almost significant for ISEI the neuroticism coefficient is markedly smaller and insignificant for CAMSIS.

	**Model 1**		**Model 2**		**Model 3**		**Model 4**		**Model 5**	
	Coeff. (SE)	p-value	Coeff. (SE)	p-value	Coeff. (SE)	p-value	Coeff. (SE)	p-value	Coeff. (SE)	p-value
Cognitive Performance	0.695*** (0.021)	0.000	0.593*** (0.029)	0.000	0.437*** (0.071)	0.000	0.446*** (0.071)	0.000	0.440*** (0.069)	0.000
ADHD			-0.292*** (0.040)	0.000	-0.360*** (0.067)	0.000	-0.412*** (0.073)	0.000	-0.392*** (0.076)	0.000
Openness					0.366** (0.129)	0.004	0.340** (0.128)	0.007	0.338** (0.127)	0.007
Risk Tolerance							0.149** (0.055)	0.008	0.143** (0.055)	0.008
Neuroticism									-0.069 (0.043)	0.102
R^2	0.483		0.558		0.670		0.688		0.693	

Supplementary Table 3. Results of multivariate genetic regression models – CAMSIS and potential mediators. Separate linear regression models with two-sided tests. Significance levels based on p-values are represented as * for p < 0.001, ** for 0.001 ≤ p < 0.01, and * for 0.01 ≤ p < 0.05.**

	Model 1		Model 2		Model 3		Model 4		Model 5	
	Coeff. (SE)	p-value	Coeff. (SE)	p-value	Coeff. (SE)	p-value	Coeff. (SE)	p-value	Coeff. (SE)	p-value
Cognitive Performance	0.680*** (0.022)	0.000	0.590*** (0.029)	0.000	0.449*** (0.069)	0.000	0.459*** (0.067)	0.000	0.450*** (0.065)	0.000
ADHD			-0.258*** (0.046)	0.000	-0.319*** (0.067)	0.000	-0.383*** (0.073)	0.000	-0.348*** (0.075)	0.000
Openness					0.331** (0.124)	0.007	0.299* (0.122)	0.013	0.296* (0.119)	0.012
Risk Tolerance							0.184*** (0.052)	0.000	0.173*** (0.051)	0.000
Neuroticism									-0.124** (0.044)	0.005
R^2	0.462		0.521		0.613		0.641		0.655	

Supplementary Table 4. Results of multivariate genetic regression models – SIOPS and potential mediators. Separate linear regression models with two-sided tests. Significance levels based on p-values are represented as *** for p < 0.001, ** for 0.001 ≤ p < 0.01, and * for 0.01 ≤ p < 0.05

	Model 1		Model 2		Model 3		Model 4		Model 5	
	Coeff. (SE)	p-value	Coeff. (SE)	p-value	Coeff. (SE)	p-value	Coeff. (SE)	p-value	Coeff. (SE)	p-value
Cognitive Performance	0.712*** (0.041)	0.000	0.616*** (0.047)	0.000	0.464*** (0.091)	0.000	0.473*** (0.092)	0.000	0.464*** (0.091)	0.000
ADHD			-0.275*** (0.058)	0.000	-0.341*** (0.086)	0.000	-0.393*** (0.091)	0.000	-0.364*** (0.095)	0.000
Openness					0.357* (0.167)	0.033	0.331* (0.169)	0.043	0.328 (0.168)	0.052
Risk Tolerance							0.149* (0.062)	0.015	0.140* (0.062)	0.024
Neuroticism									-0.103 (0.055)	0.061
R^2	0.507		0.573		0.680		0.699		0.708	

Supplementary Table 5. Results of multivariate genetic regression models – ISEI and potential mediators. Separate linear regression models with two-sided tests. Significance levels based on p-values are represented as *** for p < 0.001, ** for 0.001 ≤ p < 0.01, and * for 0.01 ≤ p < 0.05

12. Direct and indirect effects

In practice, polygenic scores might capture direct as well as (potentially noncausal) indirect genetic effects, the latter attenuating the genomic signal once the analysis is restricted to families,[49,50] reducing the utility for many practical purposes (i.e., Raben et al. 2021).[51] In particular, traits related to socioeconomic status tend to show a reduction of effect sizes in designs that allow to distinguish between direct and indirect effects.[52,53] To gauge the magnitude of these indirect effects, three designs were used.

12.1 Parental Control design

Using the genotyped subsample of the NCDS, we extended the out-of-sample prediction outlined in 8.2 by restricting our data to all respondents for whom information of the paternal occupation at age 11 was available, again pooled all waves beginning at age 33 (N = 2,988, 2,972, 2,897, 2,746, 2,369 for CAMSIS and 3,019, 2,959, ,2890, 2,742, 2,363 at age 33, 42, 46, 50, 55 for ISEI/SIOPS) and fitted a linear model with and without controlling for parental SES (operationalized as paternal occupational status at age 11 - using highest parental education lead to similar results) after controlling for age, sex and the first 10 principal components. We then computed the ratio of the PGS coefficient from both models. Results are shown in Supplementary Table 6 below.

Measure	N	Ratio (PRSice-2)	Ratio (SBayesR)	Ratio (SBayesR+MTAG)
CAMSIS	18,086	0.733	0.75	0.791
ISEI	18,093	0.707	0.743	0.785
SIOPS	18,093	0.703	0.753	0.798

Supplementary Table 6. Effect size reduction when controlling for parental SES

12.2 Adoption design

We conducted another GWAS analysis for occupational status, this time omitting the 3,398 (SIOPS, ISEI) to 3,414 (CAMSIS) participants of British-European genetic ancestry from the UK Biobank who reported being adopted and had available occupational data. We used MTAG again (SIOPS, ISEI, CAMSIS, income, and education, excluding the adoptee sample) to enhance our discovery and SBayesR to optimize the PGS's predictive capacity. In comparison to our best-performing PGS, effect sizes reduced 23.3%, 22.6%, and 27.3% respectively (R^2= 0.043, 0.031, 0.027 for CAMSIS, ISEI, SIOPS).

12.3 Sibling design

In order to identify direct genetic effects, we use the sibship sample from UK Biobank and compute fixed effects, subtracting family means from dependent and independent variables. Figure 5 in the main text displays the attenuation of the within-family signal measured by the ratio of the standardized beta coefficient of the respective best performing polygenic score

from 8.2 in a model with family fixed effects to a baseline model without fixed effects. In both cases models control for age, gender and 10 principal components on 24,579 individuals for CAMSIS, 24,472 for ISEI and SIOPS, 36,265 for education and 31,851 for income. Confidence intervals were obtained using bootstrap.

12.4 The sibling design and assortative mating

A possible explanation for the discrepancy between within-family estimates of SNP effects and GWAS estimates derived from unrelated individuals' samples could be the phenomenon of assortative mating. When the phenotypes of parents exhibit a correlation, it can result in the creation of long-range linkage disequilibrium among SNPs, even extending across different chromosomes. The subsequent increase in genetic variation in the population is not mirrored within family, leading to a reduction of effects between siblings.

Lee, Wedow, Okbay et al. (2018)[3] derive an approximation of the ratio of effect sizes for a causal SNP *j* in the population, $\beta_{BF,j}$, and within a sibship, $\beta_{WF,j}$ that can be expected as a result of a phenotypical spousal correlation *r*, when the number of loci *M* goes to infinity:

$$\lim_{M\to\infty} \frac{\beta_{WF,j}}{\beta_{BF,j}} = 1 - r\frac{(h^2-h^4 r)}{1-h^4 r} ,$$

where h^2 denotes the narrow sense heritability under assortative mating. Assuming a PGS to be a noisy measure of effect size weighted alleles of independent causal SNPs (or their LD-based proxies), this formula should provide an approximation to the expected PGS-effect reduction under the assumption of spousal phenotypic assortment, that can be estimated by plugging in plausible values for h^2 and *r* from the literature. The former can be found in the recent behavior genetics literature: Hootegem et al. (2023)[54] estimate the narrow sense heritability of SIOPS in Norway to be 0.38, Erola et al. (2022)[55] h^2 for ISEI in Finland as 0.42 and Marks (2017)[56] in Australia as 0.37.

Turning to *r*, Clark and Cummins (2022)[57] recently estimated the degree of assortative mating on occupational status in England from 1754-2021. Using a new database of 1.7 million marriage records they found it to be remarkably high: Correcting for measurement error, the groom-bride correlation equaled $r = 0.8$ with little to no variation over time.

Assuming $h^2 = 0.4$ and $r = 0.8$, $\lim_{M\to\infty} \frac{\beta_{WF,j}}{\beta_{BF,j}} = 0.75$. Then, assortative mating would explain a 25% effect reduction.

However, assortative mating can lead to downward bias in heritability estimates from twin models (Wolfram and Morris, 2023)[58] and notably only Erola et al. (2022)[55] correct for this, though only by using the phenotypic spousal correlation on educational attainment, so that even higher expected effect reductions (0.7 if $h^2 = 0.5$) might be plausible.

We further extended our investigation in order to quantify the role of assortative mating in our estimates by analyzing couples in the UK Biobank. To assess assortative mating on occupational status, we reconstruct couples according to the standards in the literature.[59] Starting with the British-European genetic ancestry subsample, household sharing information was used to

extract pairs of individuals who (a) report living with their spouse, (b) report the same length of time living in the house, (c) report the same number of occupants in the household, (d) report the same number of vehicles, (e) report the same accommodation type and rental status, (f) have identical home coordinates (rounded to the nearest km), (g) are registered with the same UK Biobank recruitment center and (h) both have available genotype data. If more than two individuals shared identical information across all variables, these individuals were excluded from analysis.

The results of phenotypic correlations are presented in the Supplementary Table 7 below. As it can be seen, of the potential spousal pairs identified in this way, 21903/21696/21696 (CAMSIS/ISEI/SIOPS) had phenotypic information on occupational status available. Significant and sizable phenotypic correlations are discernible, highest for CAMSIS (0.331, 95% CI 0.319 - 0.342), lower for ISEI (0.235, 95% CI 0.223 - 0.248) and SIOPS (0.224, 95% CI 0.211 - 0.237).

Outcome	Corr	CI (lower)	CI (upper)	N
CAMSIS	0.3305987	0.3187508	0.3423432	21,903
ISEI	0.2355167	0.2229088	0.2480458	21,696
SIOPS	0.2239975	0.2113209	0.2365988	21,696

Supplementary Table 7. Spousal correlations of occupational status scores.

For 9074 spousal pairs of British-European genetic ancestry in the UKB, no information on occupation for either partner is available. To guard against overfitting, we use these respondents, which have not been part of our GWAS to compute the correlation of polygenic scores. Under the assumption of direct assortment on the phenotype of occupational status, the correlation of the full genetic components of spouses should be equal to:

$$r_{geno,spouses} = h^2 * r_{pheno,spouses}$$

As a polygenic score represents only a noisy proxy of this genetic component, the association diminishes to:

$$r_{pgs,spouses} = r_{pgs}^2 * r_{pheno,spouses}$$

Using the validation performance within UKB for the three occupational status metrics, the expected correlation under phenotypic assortment for the best non-MTAG PGS is therefore 0.019 (CAMSIS), 0.009 (ISEI) and 0.008 (SIOPS). However, the observed correlation between spousal PGS exceeds the expected correlation substantially, closely mirroring and even exceeding earlier findings for educational attainment.[60]Accordingly, we found further support that assortative mating explains at least 25% effect reduction.

Outcome	Corr	CI (lower)	CI (upper)	N
CAMSIS	0.11544624	0.09509622	0.1356998	9,074
ISEI	0.08937277	0.06892360	0.1097469	9,074
SIOPS	0.08797762	0.06752393	0.1083574	9,074

Supplementary Table 8. Spousal polygenic scores correlations of occupational status scores. PGS calculated with SBayesR weights

13. Polygenic score prediction over the life course

For SIOPS, ISEI and CAMSIS, the incremental R-square was calculated by looking at the increase in variance explained when adding the corresponding PGS to a regression including sex and 10 principal components on either of the three occupational status metrics for all five available measurements (Supplementary Figure 77). We used the genotyped subsample of the National Child Development Study (NCDS) for these analyses and phenotypic data at ages 33, 42, 46, 50 and 55. As in the pooled case, prediction for all time points highest for CAMSIS, with comparable performance for ISEI and SIOPS. Again, SBayesR outperforms PRSice2 and is furthermore improved by the combination with an MTAG predictor (using the same construction as specified in the pooled case). Highest incremental R^2-values are achieved at age 33 (up to 0.099, SE = 0.0076 for CAMSIS) and decrease minimally up to age 55 (0.93, SE = 0.0081 for CAMSIS).

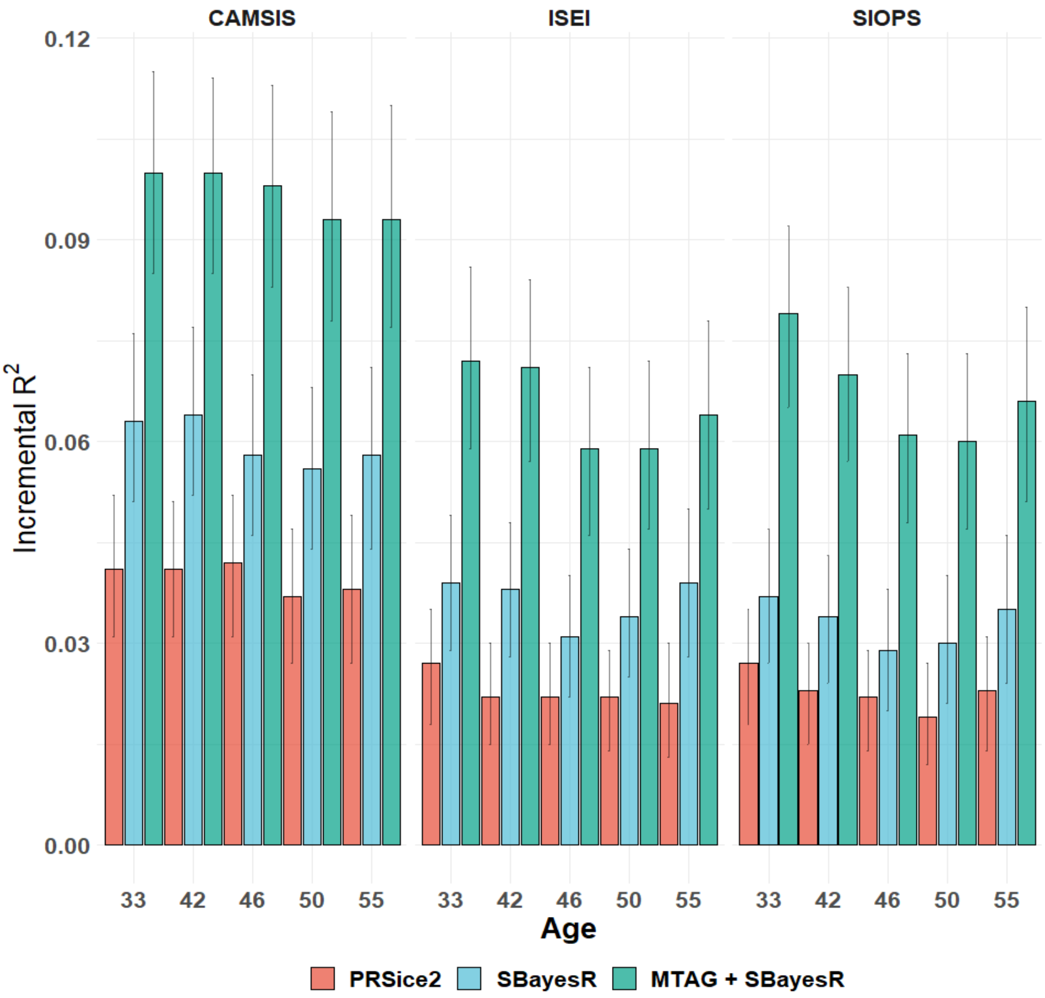

Supplementary Figure 7. Incremental R-square of polygenic score predictions of occupational status over the life course, NCDS. Bars denote 95% confidence intervals. N = 5,389; 5,312; 5,211; 4,902; 4,263 for CAMSIS at age 33, 42, 46, 50 and 55, N = 5,449; 5,293; 5,197; 4,892; 4,252 for ISEI/SIOPS. See Supplementary Table 9 for numerical values underlying this figure.

CAMSIS									
	PGS type: PRSice2			PGS type: SBayesR			PGS type: MTAG+SBayesR		
Age	Mean	Lower CI	Upper CI	Mean	Lower CI	Upper CI	Mean	Lower CI	Upper CI
33	0.041	0.031	0.052	0.063	0.051	0.076	0.099	0.085	0.115
42	0.041	0.031	0.051	0.064	0.052	0.077	0.099	0.085	0.114
46	0.042	0.031	0.052	0.058	0.046	0.070	0.098	0.083	0.113
50	0.037	0.027	0.047	0.056	0.044	0.068	0.093	0.078	0.109
55	0.038	0.027	0.049	0.058	0.044	0.071	0.093	0.077	0.110
ISEI									
33	0.027	0.018	0.035	0.039	0.029	0.049	0.072	0.059	0.086
42	0.022	0.015	0.030	0.038	0.028	0.048	0.071	0.057	0.084
46	0.022	0.015	0.030	0.031	0.022	0.040	0.059	0.046	0.071
50	0.022	0.014	0.029	0.034	0.025	0.044	0.059	0.047	0.072
55	0.021	0.013	0.030	0.039	0.028	0.050	0.064	0.050	0.078
SIOPS									
33	0.027	0.018	0.035	0.037	0.027	0.047	0.079	0.065	0.092
42	0.023	0.015	0.030	0.034	0.024	0.043	0.070	0.057	0.083
46	0.022	0.014	0.029	0.029	0.020	0.038	0.061	0.048	0.073
50	0.019	0.012	0.027	0.030	0.021	0.040	0.060	0.047	0.073
55	0.023	0.014	0.031	0.035	0.024	0.046	0.066	0.051	0.080

Supplementary Table 9. Incremental R-square of polygenic score predictions of occupational status over the life course, NCDS (95% Cis). N = 5,389; 5,312; 5,211; 4,902; 4,263 for CAMSIS at age 33, 42, 46, 50 and 55, N = 5,449; 5,293; 5,197; 4,892; 4,252 for ISEI/SIOPS.

14. Polygenic scores and the intergenerational transmission of occupational status

In addition to the various measures of occupational status over the life course, we also have information on the paternal occupation at age 11 in the NCDS data. The phenotypical correlation between paternal and offspring occupational status at the various ages for all three measures is substantial (~r=0.3). However, while often interpreted as a purely social measure of the intergenerational transmission of occupational status, it might be confounded by shared genetic potential between father and child.

Using an approach first proposed by Tucker-Drob (2017),[40] we investigate the following scenarios. Specifically, what share of the intergenerational correlation for each of the three metrics at the beginning, middle and end of the career is confounded by the corresponding polygenic score if we assume that it only explains the amount of variance it does (1) or (2) it explains the full SNP-heritability? In the first case, a small but significant amount of confounding is observed for all metrics at all ages. In the second scenario 24-38% of the

intergenerational correlation is confounded by genetic factors. Figure 8 in the main text displays the full results of these analyses.

15. Polygenic score mediation by occupational aspirations and psychological traits

While we already showed that psychological traits from the cognitive and noncognitive domain explain more than 70% of the SNP-heritability in occupational status, the NCDS data allows for the study of potential pathways from polygenic scores to occupational status using phenotypic measures of potential mediators.

15.1 Description of mediators

Occupational Aspirations: Respondents were asked at age 11 about the type of job they would like to do in the future (variable n2771). These occupations were mapped to the 1970 Classification of Occupations (variable name: co70) which was the current statistical standard in 1974 when the cohort members were interviewed. Using the mapping to co70 provided by the CAMSIS-project, a measure of occupational status was then assigned to each occupation.

Cognitive Ability: An array of tests conducted at age 11 was used to operationalize verbal ability, nonverbal ability, reading ability and mathematical ability. The associated variables are given in Table 10. We used these variables to create a measure of general cognitive ability by means of factor analysis. A 1-factor EFA was fitted using the psych package. All items show high loadings on a general ability factor and together explain more than 75% of the variance. We extract the factor scores from this model as our measure of general intelligence.

Outcome	Loading
Verbal score on general ability test (n914)	0.928
Non-verbal score on gen ability test (n917)	0.84
Reading comprehension test score (n923)	0.813
Mathematics test score (n926)	0.882
Proportion of Variance Explained	0.751

Supplementary 10. Variables used to construct general cognitive ability measure with corresponding loadings.

Scholastic Motivation: Scholastic motivation was measured by a battery of self-assessed items in Sweep 3 (age 16) using a five-point Likert-scale expressing agreement with a set of statements. The associated variables are displayed in Supplementary Table 11. A one-factor solution (based on polychoric correlations, given the ordinal nature of the data) once more fits the data well (53.7% variance explained).

Outcome	Loading
I do not like school (n2721)	0.822
School waste of time (n2716)	0.759
Homework is a bore (n2718)	0.673
I never take work seriously (n2720)	0.667
Proportion of Variance Explained	0.537

Supplementary Table 11. Variables used to construct scholastic motivation measure with corresponding loadings.

Externalizing Behavior: A wide range of items at age 16 taken from the Rutter scale was used to construct measures of externalizing behavior. Each represents agreement of a teacher with a statement on a scale from one ("Does not apply") to three ("Certainly applies") to a statement concerning the child's behavior during the past 12 months (Supplementary Table 12). All items show high loadings on a single factor in an EFA with polychoric correlations (72.3% variance explained) whose results were used to generate factor scores of externalizing behavior.

Outcome	Loading
Is often disobedient (n2310)	0.925
Frequently fights, very quarrelsome (n2300)	0.898
Often tells lies (n2314)	0.888
Destroys, damages own, others property (n2299)	0.883
Bullies other children (n2321)	0.882
Resentful, aggressive when corrected (n2320)	0.861
Restless, difficulty staying seated (n2296)	0.826
Irritable, touchy, flies off the handle (n230)	0.821

Cannot settle more than a few moments (n2311)	0.813
Squirmy, fidgety (n2298)	0.789
Has stolen at least once in past year (n2315)	0.749
Proportion of Variance Explained	0.723

Supplementary Table 12. Variables used to construct externalizing behavior measure with corresponding loadings.

Internalizing Behavior: A further subset of items (Supplementary Table 13) collected at age 16 was used to construct measures of internalizing behavior. Each again represents agreement of a teacher with a statement on a scale from one ("Does not apply") to three ("Certainly applies") to a statement concerning the child's behavior during the past 12 months. Once more, all items show high loadings on a single factor in an EFA with polychoric correlations (53.7% variance explained) whose results were used to generate factor scores of internalizing behavior.

Health: We used the earliest available measure of general health, respondent's description of their own health (age 23, variable n5739) on a scale from 1 ("poor") to 4 ("excellent").

Outcome	Loading
Often appears miserable, unhappy, etc (n2305)	0.851
Tears on arrival, refusal to enter school (n2318)	0.742
Often worries about many things (n2302)	0.728
Fearful of new situations & things (n2312)	0.703
Tends to be on own (n2303)	0.634
Proportion of Variance Explained	0.54

Supplementary Table 13. Variables used to construct internalising behavior measure with corresponding loadings.

15.2 Results

We ran mediation models from PGS to phenotypic occupational status, investigating mediation by the above specified variables. As with the genetic regression models, the largest share of

the association is explained by cognitive ability. However, for all measures and ages, a significant share of the PGS (6.5-11%, Figure 7 in the main text) is mediated by aspirations.

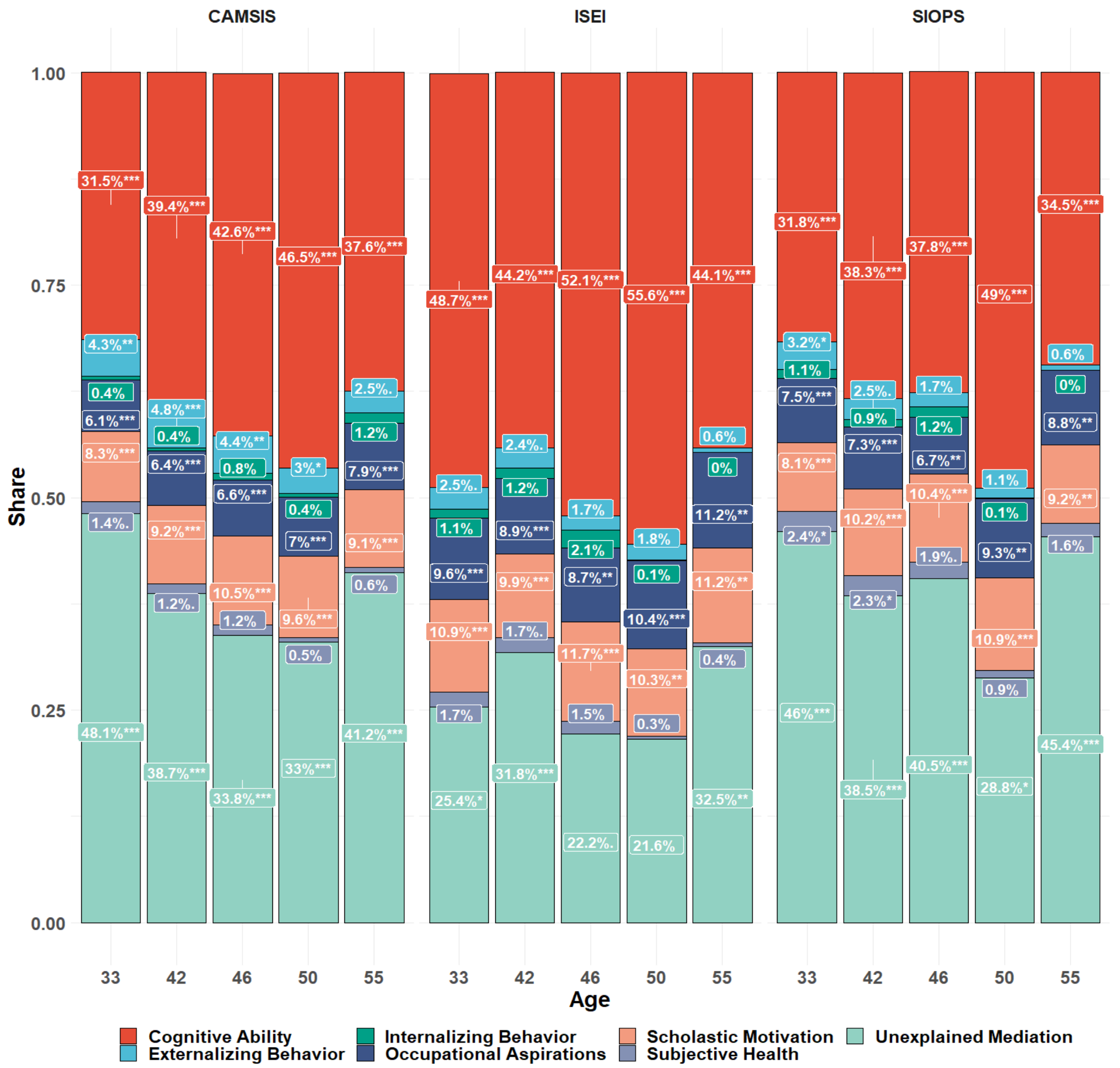

*Supplementary Figure 8. Mediation results of polygenic prediction of occupational status (controlling for parental SES), NCDS. N = 3,169; 3,111; 3,075; 2,881; 2,499 for CAMSIS at age 33, 42, 46, 50, 55 and 3,196; 3,100; 3,068; 2,878; 2,494 for SIOPS/ISEI. Stars indicate the significance level based on P values (that is, no star for not significant: P>0.05, * nominally significant: 0.01 ≤ P<0.05, ** significant at 0.001 ≤ P < 0.01, *** significant at P < 0.001.*

To control for bias from indirect genetic effects in population estimates, we replicate these analyses controlling for parental socioeconomic status, determined by paternal occupational status at age 11. We find no substantial differences in our results (Supplementary Figure 8). Whilst the total effect's size decreases, as documented in Supplementary Information 15, the relative contributions of mediators largely persist, with only minor reductions in the mediating

effect of cognitive ability accompanied by slight increases in the share of unexplained mediation.

16. Polygenic scores and occupational status trajectories throughout the careers

16.1 Data Preparation

To investigate occupational status trajectories over the life course, we relied on the NCDS activity histories dataset,[61] a rich longitudinal compilation that captures the essence of work and non-work activities from the cohort's school-leaving age onwards. Spanning multiple sweeps, this dataset provides detailed insights into the myriad activities the respondents were engaged in on up to age 55.

To construct a comprehensive view of each cohort member's career progression, each entry in the dataset was parsed to extract employment status information, the start and end dates of the role, and associated occupational codes. Non-employment related spells were removed.

In earlier sweeps of the NCDS, occupations were coded using the SOC90 system, while later sweeps transitioned to SOC2000. To create a unified framework, we used a mapping between the SOC90 and ISCO88 codes. Similarly, a mapping was used between SOC2000 and ISCO88, ensuring a seamless integration across all datasets. To ISCO-88, SIOPS and ISEI could be mapped, while direct CAMSIS mappings provided by the CAMSIS project to SOC90 and SOC2000 were used. The CAMSIS mappings also made use of information concerning employment status and sex.

Recognizing the gaps in our longitudinal dataset, either due to nonresponse or because the observed spells at the time point in question were not occupation-related, we employed linear interpolation of CAMSIS, ISEI and SIOPS, to fill in missing values between two occupation-related spells. Our results are robust to the removal of the extrapolated values.

16.2 Analysis

With our enriched dataset, we investigated the occupational status trajectories against the first 30 years of each participant's career, focusing on two particular analyses:

Socio-Educational and Sex Impact: For each occupational status measure, we assessed the evolution of occupational status over the first 30 years of a career. These trajectories were delineated by PGS quintiles, socio-educational backgrounds, and gender.

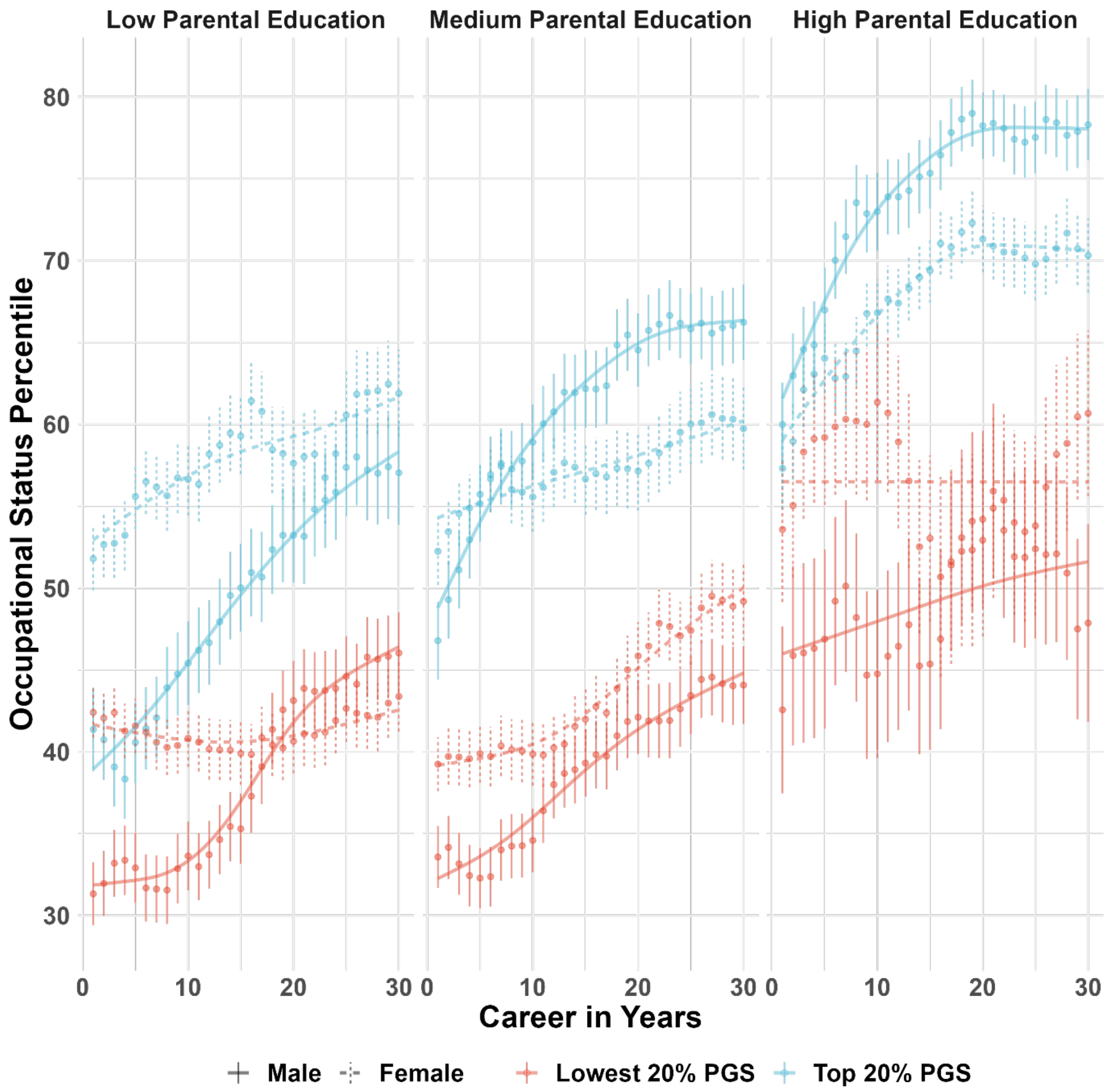

Supplementary Figure 9. Mean Percentile of the occupational status (CAMSIS) distribution across the career stratified by sex, parental education and the CAMSIS PGS. N = 201,939 time points from 5,475 individuals. Parental education measured as Low = No Qualifications, Medium = Lower Secondary, High = Upper Secondary/Degree.

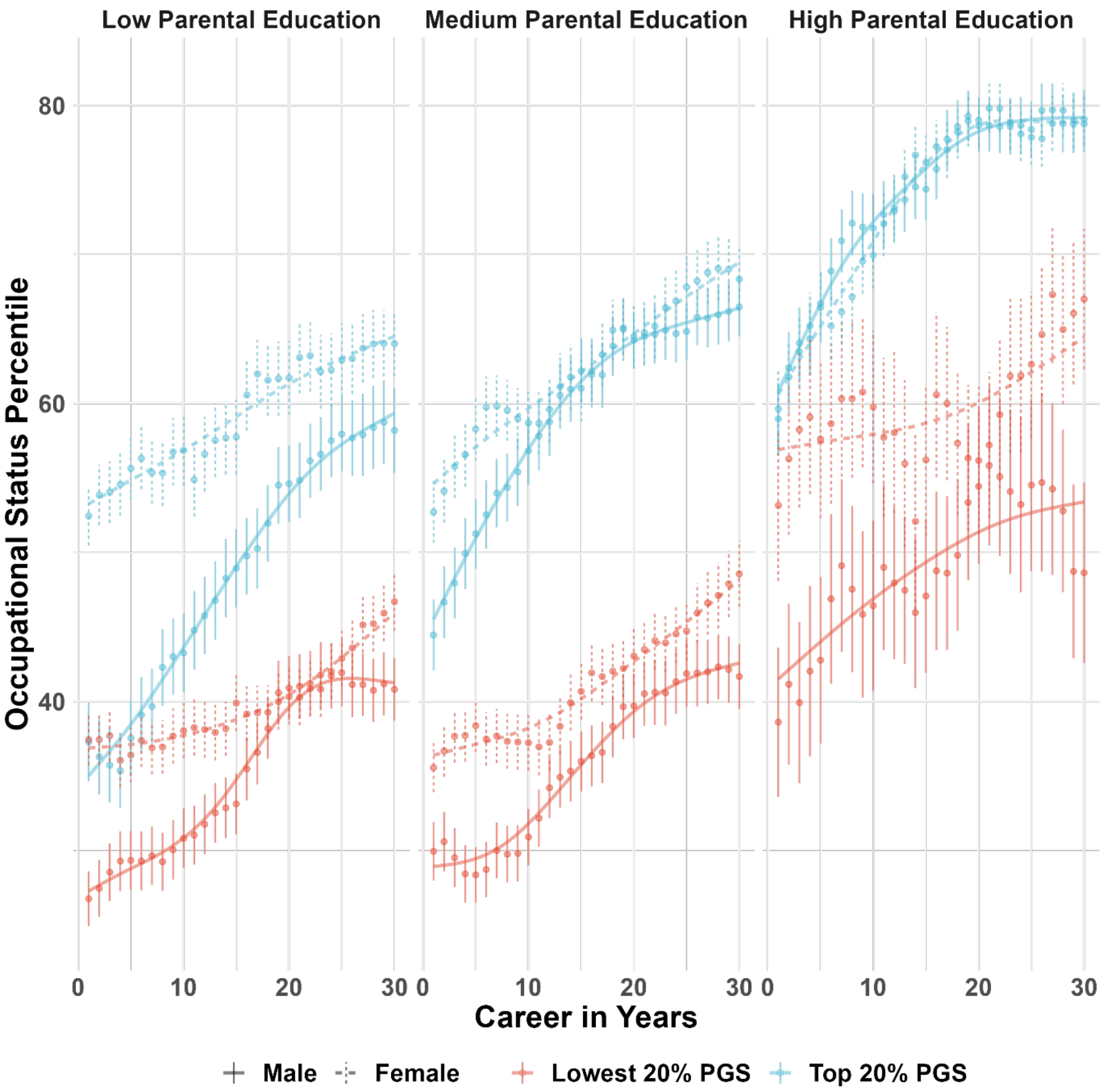

Supplementary Figure 10. Mean Percentile of the occupational status (ISEI) distribution across the career stratified by sex, parental education and the ISEI PGS. N = 201,939 time points from 5,475 individuals. Parental education measured as Low = No Qualifications, Medium = Lower Secondary, High = Upper Secondary/Degree.

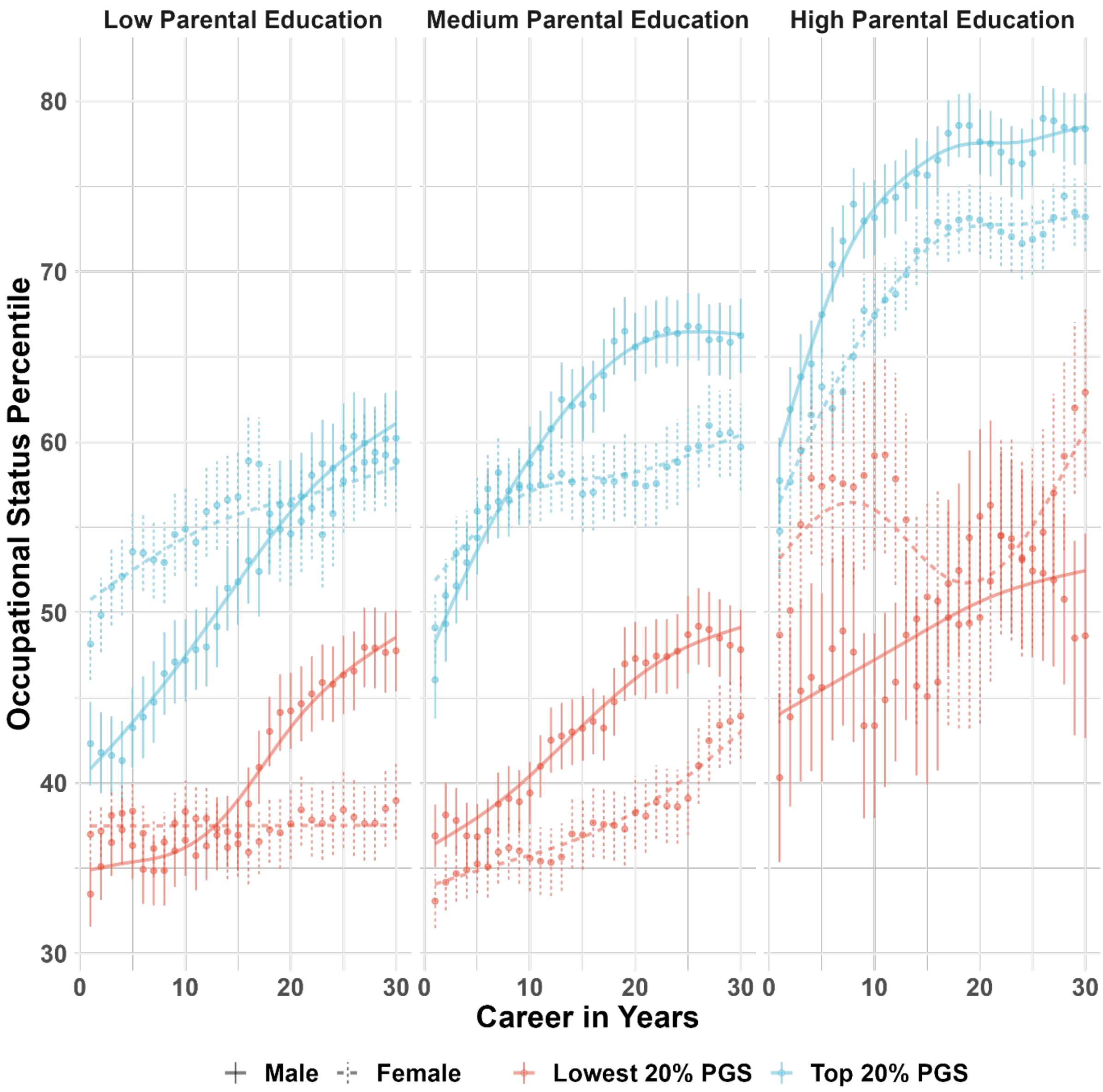

Supplementary Figure 11. Mean Percentile of the occupational status (SIOPS) distribution across the career stratified by sex, parental education and the SIOPS PGS. N = 201,939 time points from 5,475 individuals. Parental education measured as Low = No Qualifications, Medium = Lower Secondary, High = Upper Secondary/Degree.

Starting Point Impact: We also evaluated the impact of the initial occupational status at the start of a career. By examining trajectories in relation to PGS quintiles, we gauged the significance of career commencement positions on subsequent progressions.

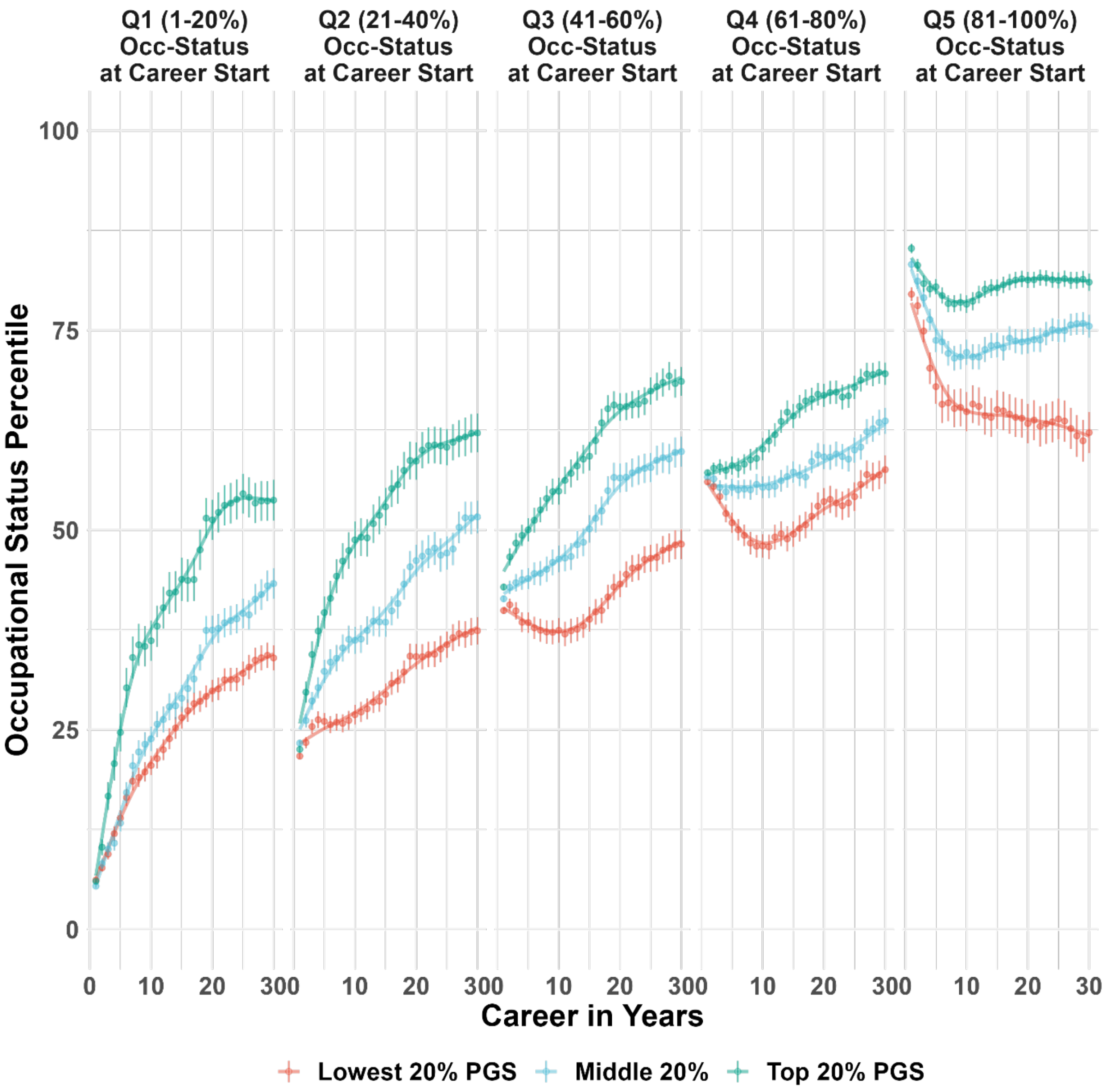

Supplementary Figure 12. Mean Percentile of the occupational status (CAMSIS) distribution across the career stratified by career start and the CAMSIS PGS. N = 201,939 time points from 5,475 individuals.

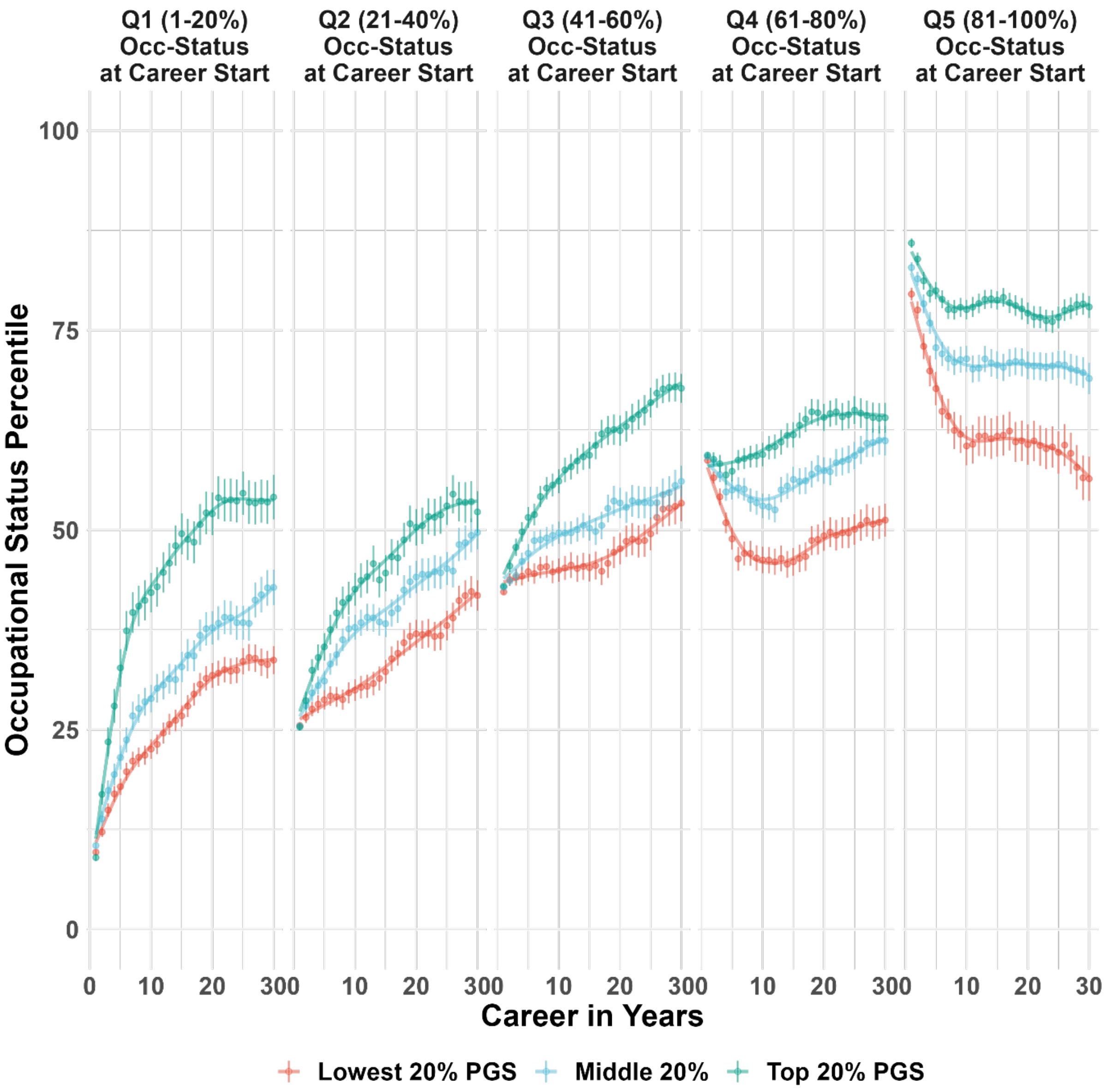

Supplementary Figure 13. Mean Percentile of the occupational status (ISEI) distribution across the career stratified by career start and the ISEI PGS. N = 201,939 time points from 5,475 individuals.

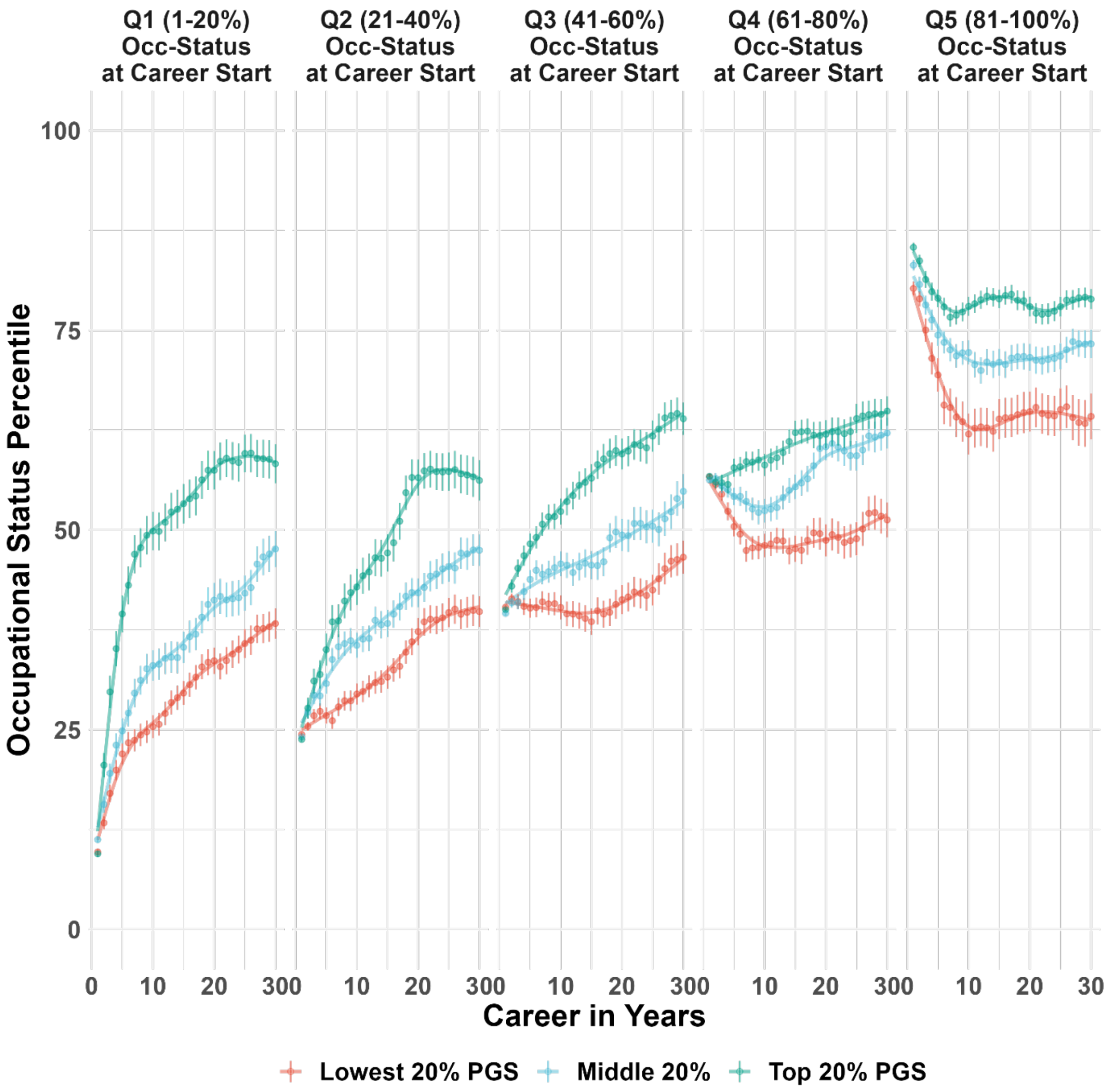

Supplementary Figure 14. Mean Percentile of the occupational status (SIOPS) distribution across the career stratified by career start and the SIOPS PGS. N = 201,939 time points from 5,475 individuals.

17. Polygenic score associations with health outcomes

In addition to the associations between the polygenic signals and occupational status, we also study its association with mental and general health outcomes. We show that the occupational status PGS has weak but robust links to health over the life course.

17.1 General health

Within the NCDS, we look at health measured at ages 23, 33, 42, 46, 50 and 55. Participants were asked to rate their general health on a scale from:

- one (excellent) to four (poor) (age 23 and 33)
- one (excellent) to five (very poor) (age 42),
- one (excellent) to five (poor) (age 46, 50 and 55)

For each time point, the outcome is treated as metric and standardized to have a mean of zero and a standard deviation of 1. We then regress it on the CAMSIS, ISEI and SIOPS PGSs, respectively, while controlling for sex and ten principal components to correct for population stratification. We find weak but highly significant positive associations of the polygenic scores in all analytic models. Results are presented in Supplementary Table 14.

	CAMSIS	ISEI	SIOPS	N
General Health at Age 23	-0.062***<br>(0.013)	-0.059***<br>(0.013)	-0.055***<br>(0.013)	5,586
General Health at Age 33	-0.1***<br>(0.013)	-0.093***<br>(0.013)	-0.088***<br>(0.013)	5,686
General Health at Age 42	-0.081***<br>(0.012)	-0.077***<br>(0.012)	-0.072***<br>(0.012)	6,233
General Health at Age 46	-0.088***<br>(0.013)	-0.088***<br>(0.013)	-0.09***<br>(0.013)	5,925
General Health at Age 50	-0.099***<br>(0.013)	-0.093***<br>(0.013)	-0.089***<br>(0.013)	5,666
General Health at Age 55	-0.111***<br>(0.013)	-0.109***<br>(0.013)	-0.107***<br>(0.013)	5,302

Supplementary Table 14. Associations between occupational status PGS and general health at various ages, controlling for sex and first 10 PCs.

17.1a Controlling for parental occupational status

Controlling for paternal occupational status at age 11 - and therefore partly for social stratification in the GWAS - does attenuate the associations; however, significant associations remain. Results are presented in Supplementary Table 15.

	CAMSIS		ISEI		SIOPS		N
	Estimate	Ratio	Estimate	Ratio	Estimate	Ratio	
General Health at Age 23	-0.049** (0.015)	0.77	-0.047** (0.015)	0.82	-0.047** (0.015)	0.80	4,050
General Health at Age 33	-0.077*** (0.015)	0.76	-0.075*** (0.015)	0.83	-0.077*** (0.015)	0.82	4,065
General Health at Age 42	-0.062*** (0.014)	0.73	-0.064*** (0.014)	0.82	-0.067*** (0.014)	0.82	4,441
General Health at Age 46	-0.053*** (0.015)	0.72	-0.065*** (0.015)	0.87	-0.069*** (0.015)	0.87	4,229
General Health at Age 50	-0.072*** (0.015)	0.75	-0.07*** (0.015)	0.81	-0.075*** (0.015)	0.82	4,067
General Health at Age 55	-0.078*** (0.016)	0.76	-0.084*** (0.016)	0.84	-0.09*** (0.016)	0.86	3,804

Supplementary Table 15. Associations between occupational status PGS and general health at various ages, controlling for father's occupational status at age 11, sex and first 10 PCs. Ratio denotes the ratio of the standardized beta-coefficient of the PGS without controlling for father's occupational status and the standardized beta-coefficient of the PGS when controlling for father's occupational status in the same sample of individuals for which paternal occupational status information was available.

17.1b Confounding of phenotypical effect of occupational status

The association between phenotypical occupational status and general health is only weakly confounded by the polygenic signal. Even when we scale the polygenic score to the SNP-heritability using the method proposed in section 12, at maximum one third of the association is confounded by the polygenic score (Supplementary Figure 15).

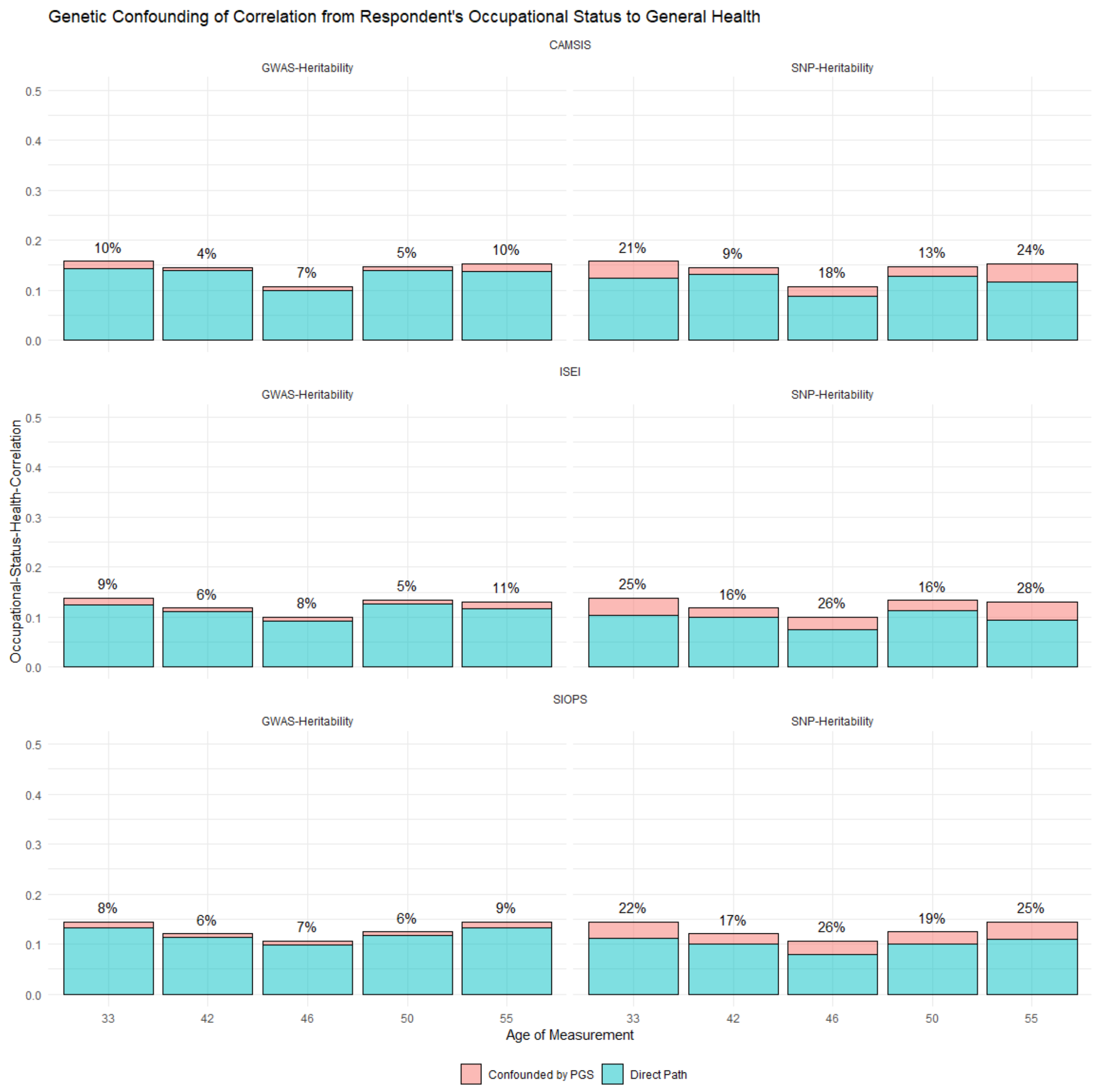

Supplementary Figure 15. Genetic confounding of correlations from respondent's occupational status to general health

17.2 Mental health

Mental health in the NCDS is measured using the Malaise Inventory,[62] a commonly used self-completion scale for the assessment of psychiatric distress. Validation studies (partially conducted within the NCDS) show high reliability and validity of the instrument.[63]

The instrument is comprised of 24 questions. Each item has the response options yes or no:

1. *Do you often have backache?*
2. *Do you feel tired most of the time?*
3. *Do you often feel miserable or depressed?*
4. *Do you often have bad headaches?*

5. *Do you often get worried about things?*
6. *Do you usually have great difficulty in falling or staying asleep?*
7. *Do you usually wake unnecessarily early in the morning?*
8. *Do you wear yourself out worrying about your health?*
9. *Do you often get in a violent rage?*
10. *Do people often annoy and irritate you?*
11. *Have you at times had twitching of the face, head, or shoulders?*
12. *Do you often suddenly become scared for no good reason?*
13. *Are you scared to be alone when there are no friends near you?*
14. *Are you easily upset or irritated?*
15. *Are you frightened of going out alone or of meeting people?*
16. *Are you constantly keyed up and jittery?*
17. *Do you suffer from indigestion?*
18. *Do you suffer from an upset stomach?*
19. *Is your appetite poor?*
20. *Does every little thing get on your nerves and wear you out?*
21. *Does your heart often race like mad?*
22. *Do you often have bad pains in your eyes?*
23. *Are you troubled with rheumatism or fibrositis?*
24. *Have you ever had a nervous breakdown?*

The Malaise Inventory was part of the survey at ages 23, 33, 42 and 50. However, at age 50, only items 3, 5, 9, 12, 14, 16, 20 and 21 were asked. We therefore restrict ourselves to these eight items in our analyses. For all four time points, composite scores are created by the means of factor analysis and subsequently standardized. Loadings on a single factor were overall very high and similar for the different time points.

For each wave, we regress the three occupational status polygenic scores separately on the factor, while controlling for sex and the first ten principal components. Here, we find weak but significant positive associations between mental health and the three PGSs. Results are presented in Supplementary Table 16.

	CAMSIS	ISEI	SIOPS	N
Mental Health at Age 23	-0.08*** (0.012)	-0.074*** (0.012)	-0.073*** (0.012)	5,545
Mental Health at Age 33	-0.054*** (0.012)	-0.05*** (0.012)	-0.049*** (0.012)	5,698
Mental Health at Age 42	-0.036** (0.012)	-0.036** (0.012)	-0.038** (0.012)	6,205
Mental Health at Age 50	-0.033* (0.014)	-0.042** (0.014)	-0.035* (0.014)	5,057

Supplementary Table 16. Associations between occupational status PGS and mental health at various ages, controlling for sex and first 10 PCs.

17.2a Controlling for parental occupational status

Supplementary Table 17 presents the results of the models where we control for paternal occupational status. Some of the results are not significant but remain directionally similar which means that parental characteristics confound the association between occupational status polygenic score and mental health.

17.2b Confounding of phenotypical effect of occupational status and mental health

The association between phenotypical occupational status and general health is only weakly confounded by the polygenic signal. Once we scale the polygenic score to the SNP-heritability, the confounding becomes stronger. Nevertheless, the overall effects are small, as seen in Supplementary Figure 16.

	CAMSIS		ISEI		SIOPS		N
	Estimate	Ratio	Estimate	Ratio	Estimate	Ratio	
Mental Health at Age 23	-0.056*** (0.014)	0.80	-0.05*** (0.014)	0.86	-0.05*** (0.014)	0.84	4,018
Mental Health at Age 33	-0.034* (0.015)	0.70	-0.027. (0.015)	0.74	-0.027. (0.015)	0.72	4,072
Mental Health at Age 42	-0.029* (0.014)	0.72	-0.029* (0.014)	0.77	-0.032* (0.014)	0.80	4,420
Mental Health at Age 50	-0.02 (0.016)	0.64	-0.032* (0.016)	0.82	-0.024 (0.016)	0.75	3,646

Supplementary Table 17. Associations between occupational status PGS and mental health at various ages, controlling for father's occupational status at age 11, sex and first 10 PCs. Ratio denotes the ratio of the standardized beta-coefficient of the PGS without controlling for father's occupational status and the standardized beta-coefficient of the PGS when controlling for father's occupational status in the same sample of individuals for which paternal occupational status information was available.

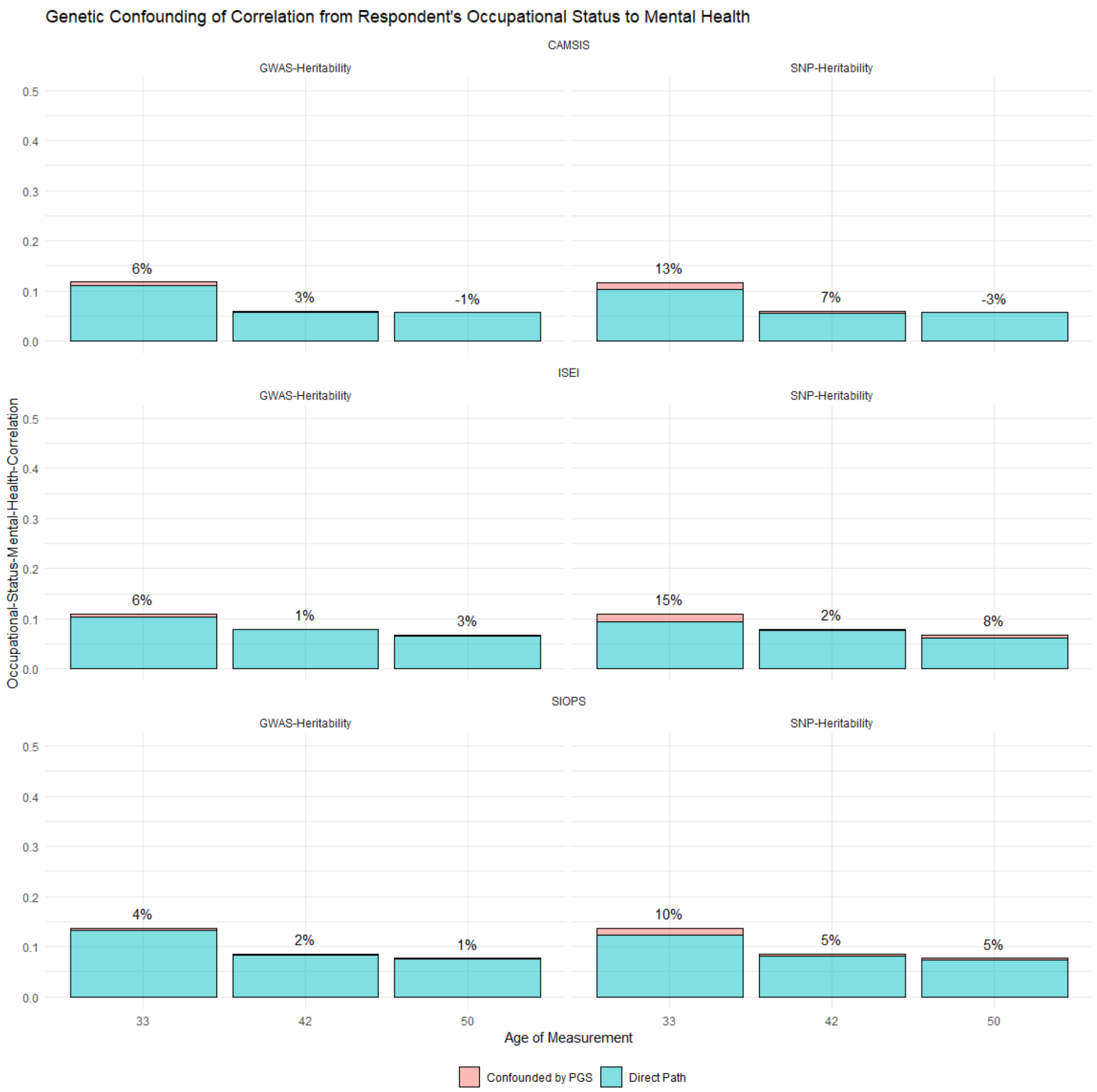

Supplementary Figure 16. Genetic confounding of correlations from respondent's occupational status to mental health

References

1. Fujishiro, K., Xu, J. & Gong, F. What does "occupation" represent as an indicator of socioeconomic status?: Exploring occupational prestige and health. *Social Science & Medicine* **71**, 2100-2107 (2010).
2. Marmot, M.G. *et al.* Health inequalities among British civil servants: the Whitehall II study. *Lancet* **337**, 1387-93 (1991).
3. Lee, J.J. *et al.* Gene discovery and polygenic prediction from a genome-wide association study of educational attainment in 1.1 million individuals. *Nature Genetics* **50**, 1112-1121 (2018).
4. Okbay, A. *et al.* Genome-wide association study identifies 74 loci associated with educational attainment. *Nature* **533**, 539-542 (2016).
5. Rietveld, C.A. *et al.* GWAS of 126,559 individuals identifies genetic variants associated with educational attainment. *Science* **340**, 1467-71 (2013).
6. Hill, W.D. *et al.* Molecular Genetic Contributions to Social Deprivation and Household Income in UK Biobank. *Current biology : CB* **26**, 3083-3089 (2016).
7. Hill, W.D. *et al.* Genome-wide analysis identifies molecular systems and 149 genetic loci associated with income. *Nature Communications* **10**, 5741 (2019).
8. Rietveld, C.A., Slob, E.A.W. & Thurik, A.R. A decade of research on the genetics of entrepreneurship: a review and view ahead. *Small Business Economics* **57**, 1303-1317 (2021).
9. Sulkava, S. *et al.* Common Genetic Variation Near Melatonin Receptor 1A Gene Linked to Job-Related Exhaustion in Shift Workers. *Sleep* **40**(2017).
10. Sulkava, S. *et al.* Genome-wide scan of job-related exhaustion with three replication studies implicate a susceptibility variant at the UST gene locus. *Human Molecular Genetics* **22**, 3363-3372 (2013).
11. Macintyre, S. The Black Report and beyond: what are the issues? *Soc Sci Med* **44**, 723-45 (1997).
12. Connelly, R., Gayle, V. & Lambert, P.S. A Review of occupation-based social classifications for social survey research. *Methodological Innovations* **9**, 2059799116638003 (2016).
13. Blishen, B.R. The Construction and Use of an Occupational Class Scale. *Canadian Journal of Economics and Political Science* **24**, 519-525 (1958).
14. Duncan, O.D. A socioeconomic index for all occupations. *Class: Critical Concepts* **1**, 388-426 (1961).
15. Ganzeboom, H.B.G., De Graaf, P.M. & Treiman, D.J. A standard international socio-economic index of occupational status. *Social Science Research* **21**, 1-56 (1992).
16. Nakao, K. & Treas, J. *The 1989 socioeconomic index of occupations: Construction from the 1989 occupational prestige scores*, (National Opinion Research Center Chicago, IL, 1992).
17. Treiman, D.J. *Occupational prestige in comparative perspective*, (Elsevier, 2013).
18. Ganzeboom, H.B. & Treiman, D.J. Internationally comparable measures of occupational status for the 1988 International Standard Classification of Occupations. *Social science research* **25**, 201-239 (1996).
19. Warner, W.L., Meeker, M. & Eells, K. Social class in America; a manual of procedure for the measurement of social status. (1949).

20. Laumann, E.O. & Guttman, L. The relative associational contiguity of occupations in an urban setting. *American sociological review*, 169-178 (1966).
21. Lambert, P. & Griffiths, D. *Social Inequalities and Occupational Stratification: Methods and concepts in the analysis of social distance*, (Springer, 2018).
22. Bycroft, C. *et al.* The UK Biobank resource with deep phenotyping and genomic data. *Nature* **562**, 203-209 (2018).
23. Sudlow, C. *et al.* UK biobank: an open access resource for identifying the causes of a wide range of complex diseases of middle and old age. *PLoS Med* **12**, e1001779 (2015).
24. Lambert, P.S. & Prandy, K. CAMSIS project webpages: Cambridge social interaction and stratification scales. (https://www.camsis.stir.ac.uk/, 2012).
25. Hermans, M. The Strat Package: Tools for Social Stratification Research. (2010).
26. Office, I.L. International Standard Classification of Occupations: ISCO-88. (1990).
27. Lambert, P.S. An illustrative guide: Using GEODE to link data from SOC-2000 to NS-SEC and other occupation-based social classifications. (Citeseer, 2007).
28. Jiang, L. *et al.* A resource-efficient tool for mixed model association analysis of large-scale data. *Nature genetics* **51**, 1749-1755 (2019).
29. Okbay, A. *et al.* Genome-wide association study identifies 74 loci associated with educational attainment. *Nature* **533**, 539-42 (2016).
30. Finucane, H.K. *et al.* Partitioning heritability by functional annotation using genome-wide association summary statistics. *Nature genetics* **47**, 1228-1235 (2015).
31. Bulik-Sullivan, B. *et al.* An atlas of genetic correlations across human diseases and traits. *Nature Genetics* **47**, 1236-1241 (2015).
32. Bulik-Sullivan, B.K. *et al.* LD Score regression distinguishes confounding from polygenicity in genome-wide association studies. *Nat Genet* **47**, 291-5 (2015).
33. Euesden, J., Lewis, C.M. & O'Reilly, P.F. PRSice: polygenic risk score software. *Bioinformatics* **31**, 1466-1468 (2015).
34. Lloyd-Jones, L.R. *et al.* Improved polygenic prediction by Bayesian multiple regression on summary statistics. *Nature Communications* **10**, 5086 (2019).
35. Turley, P. *et al.* Multi-trait analysis of genome-wide association summary statistics using MTAG. *Nature Genetics* **50**, 229-237 (2018).
36. Ko, H. *et al.* Genome-wide association study of occupational attainment as a proxy for cognitive reserve. *Brain* **145**, 1436-1448 (2022).
37. Grotzinger, A.D. *et al.* Genomic structural equation modelling provides insights into the multivariate genetic architecture of complex traits. *Nature human behaviour* **3**, 513-525 (2019).
38. Spinath, F.M. & Bleidorn, W. The new look of behavioral genetics in social inequality: Gene-environment interplay and life chances. *Journal of Personality* (2017).
39. Krapohl, E. *et al.* The high heritability of educational achievement reflects many genetically influenced traits, not just intelligence. *Proceedings of the national academy of sciences* **111**, 15273-15278 (2014).
40. Briley, D.A. & Tucker-Drob, E.M. Comparing the developmental genetics of cognition and personality over the life span. *Journal of Personality* **85**, 51-64 (2017).
41. Greven, C.U., Harlaar, N., Kovas, Y., Chamorro-Premuzic, T. & Plomin, R. More than just IQ: School achievement is predicted by self-perceived abilities—But for genetic rather than environmental reasons. *Psychological Science* **20**, 753-762 (2009).

42. Klassen, L., Eifler, E.F., Hufer, A. & Riemann, R. Why do people differ in their achievement motivation? A nuclear twin family study. *Primenjena psihologija* **11**, 433-450 (2018).
43. Willems, Y., Boesen, N., Li, J., Finkenauer, C. & Bartels, M. The heritability of self-control: A meta-analysis. *Neuroscience & Biobehavioral Reviews* **100**, 324-334 (2019).
44. Demontis, D. *et al.* Discovery of the first genome-wide significant risk loci for attention deficit/hyperactivity disorder. *Nature genetics* **51**, 63-75 (2019).
45. Loh, P.-R., Kichaev, G., Gazal, S., Schoech, A.P. & Price, A.L. Mixed-model association for biobank-scale datasets. *Nature genetics* **50**, 906-908 (2018).
46. Karlsson Linnér, R. *et al.* Genome-wide association analyses of risk tolerance and risky behaviors in over 1 million individuals identify hundreds of loci and shared genetic influences. *Nature genetics* **51**, 245-257 (2019).
47. Hill, W.D. *et al.* Genetic contributions to two special factors of neuroticism are associated with affluence, higher intelligence, better health, and longer life. *Molecular psychiatry* **25**, 3034-3052 (2020).
48. Nieuwboer, H.A., Pool, R., Dolan, C.V., Boomsma, D.I. & Nivard, M.G. GWIS: genome-wide inferred statistics for functions of multiple phenotypes. *The American Journal of Human Genetics* **99**, 917-927 (2016).
49. Bates, T.C. *et al.* The nature of nurture: Using a virtual-parent design to test parenting effects on children's educational attainment in genotyped families. *Twin Research and Human Genetics* **21**, 73-83 (2018).
50. Kong, A. *et al.* The nature of nurture: Effects of parental genotypes. *Science* **359**, 424-428 (2018).
51. Raben, T.G., Lello, L., Widen, E. & Hsu, S.D. From Genotype to Phenotype: polygenic prediction of complex human traits. *arXiv preprint arXiv:2101.05870* (2021).
52. Selzam, S. *et al.* Comparing within-and between-family polygenic score prediction. *The American Journal of Human Genetics* **105**, 351-363 (2019).
53. Wang, B. *et al.* Genetic nurture effects on education: a systematic review and meta-analysis. *bioRxiv* (2021).
54. Van Hootegem, A., Rogne, A.F. & Lyngstad, T.H. Heritability of class: Implications for theory and research on social mobility. (2023).
55. Erola, J., Lehti, H., Baier, T. & Karhula, A. Socioeconomic background and gene–environment interplay in social stratification across the early life course. *European Sociological Review* **38**, 1-17 (2022).
56. Marks, G.N. The Contribution of Genes and the Environment to Educational and Socioeconomic Attainments in Australia. *Twin Research and Human Genetics* **20**, 281-289 (2017).
57. Clark, G. & Cummins, N. Assortative Mating and the Industrial Revolution: England, 1754-2021. (2022).
58. Wolfram, T. & Morris, D. Conventional twin studies overestimate the environmental differences between families relevant to educational attainment. (2022).
59. Howe, L.J. *et al.* Genetic evidence for assortative mating on alcohol consumption in the UK Biobank. *Nature Communications* **10**, 5039 (2019).
60. Okbay, A. *et al.* Polygenic prediction of educational attainment within and between families from genome-wide association analyses in 3 million individuals. *Nature genetics* **54**, 437-449 (2022).

61. University of London, I.o.E., Centre for Longitudinal Studies. National Child Development Study: Activity Histories, 1974–2013. (UK Data Service, 2020).
62. Rutter, M., Tizard, J. & Whitmore, K. *Education, health and behaviour: psychological and medical study of childhood development*, (Longman Group Limited, 1970).
63. Rodgers, B., Pickles, A., Power, C., Collishaw, S. & Maughan, B. Validity of the Malaise Inventory in general population samples. *Social psychiatry and psychiatric epidemiology* **34**, 333-341 (1999).
