# Supplementary material for: Polygenic prediction of occupational status GWAS elucidates genetic and environmental interplay in intergenerational transmission, careers and health in UK Biobank
Source: Nat Hum Behav. 2024 Dec 23;9(2):391–405. doi: 10.1038/s41562-024-02076-3 (PMC11860221; doi:10.1038/s41562-024-02076-3)
Supplement: Supplementary file 2 — Reporting Summary [file 41562_2024_2076_MOESM2_ESM.pdf]

nature portfolio

Corresponding author(s): Evelina T. Akimova
Tobias Wolfram

Last updated by author(s): 02/08/2024

Reporting Summary

Nature Portfolio wishes to improve the reproducibility of the work that we publish. This form provides structure for consistency and transparency in reporting. For further information on Nature Portfolio policies, see our Editorial Policies and the Editorial Policy Checklist.

Statistics

For all statistical analyses, confirm that the following items are present in the figure legend, table legend, main text, or Methods section.

n/a	Confirmed	
☐	☒	The exact sample size (*n*) for each experimental group/condition, given as a discrete number and unit of measurement
☐	☒	A statement on whether measurements were taken from distinct samples or whether the same sample was measured repeatedly
☐	☒	The statistical test(s) used AND whether they are one- or two-sided<br>*Only common tests should be described solely by name; describe more complex techniques in the Methods section.*
☐	☒	A description of all covariates tested
☐	☒	A description of any assumptions or corrections, such as tests of normality and adjustment for multiple comparisons
☐	☒	A full description of the statistical parameters including central tendency (e.g. means) or other basic estimates (e.g. regression coefficient) AND variation (e.g. standard deviation) or associated estimates of uncertainty (e.g. confidence intervals)
☐	☒	For null hypothesis testing, the test statistic (e.g. *F*, *t*, *r*) with confidence intervals, effect sizes, degrees of freedom and *P* value noted<br>*Give P values as exact values whenever suitable.*
☐	☒	For Bayesian analysis, information on the choice of priors and Markov chain Monte Carlo settings
☒	☐	For hierarchical and complex designs, identification of the appropriate level for tests and full reporting of outcomes
☐	☒	Estimates of effect sizes (e.g. Cohen's *d*, Pearson's *r*), indicating how they were calculated

Our web collection on statistics for biologists contains articles on many of the points above.

Software and code

Policy information about availability of computer code

Data collection	No software used for data collection.

Data analysis	Analytical methods and software are described in the Methods section and in the Supplementary Information. Software used:<br>PLINK v1.9 (https://www.cog-genomics.org/plink/1.9/)<br>PLINK v2 (https://www.cog-genomics.org/plink/2.0/)<br>LDSC v1.0.1 (https://github.com/bulik/ldsc)<br>R versions v4.2.0, v4.1.2 (https://www.r-project.org)<br>Python v2.7, v3.8.4, v3.9.15 (https:www.anaconda.org)<br>FastGWA (https://yanglab.westlake.edu.cn/software/gcta/#fastGWA)<br>FUMA v1.5.2 (https://fuma.ctglab.nl)<br>MAGMA v1.10 (https://cncr.nl/research/magma/)<br>MTAG v0.9.0 (https:www.github.com/JonJala/mtag)<br>GenomicSEM v0.0.5 (https://github.com/GenomicSEM/GenomicSEM)<br>SBayesR v2.03 (https://cnsgenomics.com/software/gctb/#Overview)<br>PRSice v2 (https://choishingwan.github.io/PRSice/)<br>R-package "lavaan" v0.6-18 (https://cran.r-project.org/web/packages/lavaan/index.html)<br>R-package "strat" v0.1 (https://cran.r-project.org/web/packages/strat/index.html)<br><br>R-package "ukbjobs" available at https://github.com/tobiaswolfram/ukbjobs, https://doi.org/10.5281/zenodo.10061205. The package allows researchers to construct CAMSIS, ISEI, and SIOPS occupational scores directly from the UK Biobank data.

For manuscripts utilizing custom algorithms or software that are central to the research but not yet described in published literature, software must be made available to editors and reviewers. We strongly encourage code deposition in a community repository (e.g. GitHub). See the Nature Portfolio guidelines for submitting code & software for further information.

Data

Policy information about availability of data

All manuscripts must include a data availability statement. This statement should provide the following information, where applicable:

- Accession codes, unique identifiers, or web links for publicly available datasets
- A description of any restrictions on data availability
- For clinical datasets or third party data, please ensure that the statement adheres to our policy

The GWAS summary statistics generated in this study are available on the GWAS catalogue website (https://www.ebi.ac.uk/gwas/) under accession codes GCST90446160, GCST90446162, GCST90446163. Access to the UK Biobank is available through: http://www.ukbiobank.ac.uk. Access to The National Child Development Study (NCDS) is available through: https://cls.ucl.ac.uk/data-access-training/. PheWAS analyses was performed using the IEU OpenGWAS project data available at: https://gwas.mrcieu.ac.uk. LDSC regression analysis was performed by using LD scores and weights available at: https://utexas.app.box.com/s/vkd36n197m8klbaio3yzoxsee6sxo11v. Analysis of the representativity of the UK Biobank with the Office of National Statistics (ONS) data was performed using publicly available ONS data which can be accessed at: https://www.ons.gov.uk/employmentandlabourmarket/peopleinwork/employmentandemployeetypes/datasets/employmentbyoccupationemp04. Source data are provided with this paper.

Research involving human participants, their data, or biological material

Policy information about studies with human participants or human data. See also policy information about sex, gender (identity/presentation), and sexual orientation and race, ethnicity and racism.

Reporting on sex and gender	All analyses were performed in sex combined models. We do not directly measure gender and accordingly do not report on it.
Reporting on race, ethnicity, or other socially relevant groupings	Our research sample contains British-European genetic ancestry only; we reflect on this in Box 1 and Discussion. We also developed an extensive FAQs along with a "key-points" section and are transparent in our use of genetic ancestry term. Genetic ancestry was determined based on principal components (PC) analysis of the genetic data. We focus on individuals of British-European genetic ancestry in order to decrease the risk of confounding due to population stratification.
Population characteristics	Population characteristics for both samples are described in the "Behavioural & social sciences study design" section below.
Recruitment	Recruitment was performed independently by UK Biobank and National Child Development Study (NCDS).
Ethics oversight	This research was conducted using the UK Biobank under application 32696 and NCDS under application GDAC_2021_16_TROPF, with ethical approval from the University of Oxford under application SOC_R2_001_C1A_21_60. Both the UK Biobank and NCDS applications were specific to the scope of this paper. For the UK Biobank approval, we received approval for a scope extension to ensure transparency, allowing us to expand from our focus on non-standard occupations to also include occupational status. Here we specified that our plan was: "to perform GWAS analysis using employment histories from the UK Biobank to construct sociologically informed measures of occupational status." We specified that we would construct sociologically informed measures of occupational status (CAMSIS, SIOPS, and ISEA) for our GWAS and noted that the analysis would be accompanied by NCDS genetic and phenotypic data. For the NCDS application, we specified not only the information mentioned above but also the set of polygenic prediction analyses. We also preregistered our analysis plan (https://osf.io/djbr2/) which was updated for replication (https://osf.io/x6va5).

Note that full information on the approval of the study protocol must also be provided in the manuscript.

Field-specific reporting

Please select the one below that is the best fit for your research. If you are not sure, read the appropriate sections before making your selection.

☐ Life sciences ☒ Behavioural & social sciences ☐ Ecological, evolutionary & environmental sciences

For a reference copy of the document with all sections, see nature.com/documents/nr-reporting-summary-flat.pdf

Behavioural & social sciences study design

All studies must disclose on these points even when the disclosure is negative.

Study description	This is a genome-wide association study (GWAS) on sociologically informed occupational status measures (ISEI, SIOPS, and CAMSIS) using the UKBiobank with multiple analytic approaches employed including genomic structural equation models (GSEM), multi-trait analysis (MTAG), sibling and adoption models (the full list of the approaches and the motivation can be found in Supplimentary Information Section 1).
Research sample	The research sample includes two data sources. First, the UK Biobank is a large-scale biomedical database and research resource, containing in-depth genetic and health information from 502,655 individuals recruited between 2006 and 2010. More information is available at: http://www.ukbiobank.ac.uk. Second, the National Child Development Study (NCDS) follows 17,000 children born in Great Britain in one week in 1958. More information is available at: https://ncds.info.<br><br>Overall, the research sample in total consists of 273,157 (130,952 males, 142,205 females) and 271,769 (130,129 males, 141,640 females) individuals for the occupational status phenotypes (CAMSIS and SIOPS/ISEI) and 353,673 (169,201 males, 184,472 females) and 404,420 (185,632 males, 218,788 females) individuals for the secondary analyses (household income and education), respectively. To validate our findings, we replicated our top hits using the genotyped subsample of the NCDS, including approximately 6,500 individuals with both genetic and phenotypic information. UK Biobank participants were between 40 and 69 years of age at the time of their recruitment between 2006 and 2010. For the NCDS, since it is a longitudinal study, observations for current occupations were pooled over all waves starting at age 33 (N = 5,389; 5,312; 5,211; 4,902; 4,263 for CAMSIS at age 33, 42, 46, 50, and 55, N = 5,449; 5,293; 5,197; 4,892; 4,252 for ISEI/SIOPS).<br><br>The rationale for using these samples is the following: the UK Biobank has the required large sample size and detailed occupational codes. We then replicated our results using the NCDS sample. This dataset was chosen because it is a similar UK cohort, which is important since previous research has demonstrated genetic variation by country and birth cohort for complex behavioral phenotypes.<br><br>The UK Biobank is not a nationally representative study; NCDS is a cohort study and representative for its respective birth cohort in the UK (born in 1958).
Sampling strategy	To obtain the largest samples possible for both discovery and replication that would cover participants of roughly the same age and with detailed occupation information from the United Kingdom.
Data collection	Data collection was performed independently by the UK Biobank and the NCDS. Both are observational studies used for secondary data analysis. Since this is not a controlled randomized study, there was no step involved equivalent to blinding.
Timing	UK Biobank and NCDS have variable data collection time-periods. UK Biobank recruited individuals between 2006 and 2010; NCDS is a cohort study and includes those born in one week in 1958.
Data exclusions	Any observations without SOC2000 occupational information were excluded; genetic and phenotypic quality controls were implemented as well (Supplimentary Information 7.2 describes them in detail). We also restricted our analytic sample to British-European genetic ancestry only. Overall, we excluded 229,462 individuals for CAMSIS analyses, 230,851 individuals for ISEI/SIOPS analyses; 148,947 and 98,200 individuals for the secondary analyses - household income and education respectively.
Non-participation	The UK Biobank response rate was 5.5%. In the NCDS, the response rate at the first sweep was 98.7%. Additionally, 25.8% have participated in all 11 sweeps, and 60.5% have taken part in 7 or more sweeps. Participants were able to select 'Prefer not to answer' options through the questionnaires in the UK Biobank and 'Refusal' options in the NCDS.
Randomization	Participants were not allocated into experimental groups.

Reporting for specific materials, systems and methods

We require information from authors about some types of materials, experimental systems and methods used in many studies. Here, indicate whether each material, system or method listed is relevant to your study. If you are not sure if a list item applies to your research, read the appropriate section before selecting a response.

Materials & experimental systems

n/a	Involved in the study
☒	☐ Antibodies
☒	☐ Eukaryotic cell lines
☒	☐ Palaeontology and archaeology
☒	☐ Animals and other organisms
☒	☐ Clinical data
☒	☐ Dual use research of concern
☒	☐ Plants

Methods

n/a	Involved in the study
☒	☐ ChIP-seq
☒	☐ Flow cytometry
☒	☐ MRI-based neuroimaging

Plants

Seed stocks

Report on the source of all seed stocks or other plant material used. If applicable, state the seed stock centre and catalogue number. If plant specimens were collected from the field, describe the collection location, date and sampling procedures.

Novel plant genotypes

Describe the methods by which all novel plant genotypes were produced. This includes those generated by transgenic approaches, gene editing, chemical/radiation-based mutagenesis and hybridization. For transgenic lines, describe the transformation method, the number of independent lines analyzed and the generation upon which experiments were performed. For gene-edited lines, describe the editor used, the endogenous sequence targeted for editing, the targeting guide RNA sequence (if applicable) and how the editor was applied.

Authentication

Describe any authentication procedures for each seed stock used or novel genotype generated. Describe any experiments used to assess the effect of a mutation and, where applicable, how potential secondary effects (e.g. second site T-DNA insertions, mosiacism, off-target gene editing) were examined.
